# The non-canonical BAF chromatin remodeling complex is a novel target of spliceosome dysregulation in *SF3B1*-mutated chronic lymphocytic leukemia

**DOI:** 10.1038/s41375-024-02379-4

**Published:** 2024-09-11

**Authors:** Daniel Hägerstrand, Blaž Oder, Diego Cortese, Ying Qu, Amrei Binzer-Panchal, Cecilia Österholm, Teresa Del Peso Santos, Leily Rabbani, Hassan Foroughi Asl, Aron Skaftason, Viktor Ljungström, August Lundholm, Maria Koutroumani, Zahra Haider, Cecilia Jylhä, John Mollstedt, Larry Mansouri, Karla Plevova, Andreas Agathangelidis, Lydia Scarfò, Marine Armand, Alice F. Muggen, Neil E. Kay, Tait Shanafelt, Davide Rossi, Lukas M. Orre, Sarka Pospisilova, Konstantin Barylyuk, Frederic Davi, Mattias Vesterlund, Anton W. Langerak, Janne Lehtiö, Paolo Ghia, Kostas Stamatopoulos, Lesley-Ann Sutton, Richard Rosenquist

**Affiliations:** 1 Department of Molecular Medicine and Surgery, Karolinska Institutet, Stockholm, Sweden; 2 Department of Immunology, Genetics and Pathology, Science for Life Laboratory, Uppsala University, Uppsala, Sweden; 3 Institute of Applied Biosciences, Centre for Research and Technology Hellas, Thessaloniki, Greece; 4 Clinical Genetics and Genomics, Karolinska University Laboratory, Karolinska University Hospital, Stockholm, Sweden; 5 Department of Internal Medicine – Hematology and Oncology, Medical Faculty, Masaryk University and University Hospital Brno, Brno, Czech Republic; 6 Institute of Medical Genetics and Genomics, Medical Faculty, Masaryk University and University Hospital Brno, Brno, Czech Republic; 7 Central European Institute of Technology, Masaryk University, Brno, Czech Republic; 8 Department of Biology, School of Science, National and Kapodistrian University of Athens, Athens, Greece; 9 Università Vita-Salute San Raffaele, Milan, Italy; 10 Division of Experimental Oncology, IRCCS, Ospedale San Raffaele, Milan, Italy; 11 Department of Hematology, Hospital Pitie-Salpetriere, Sorbonne University, Paris, France; 12 Department of Immunology, Erasmus MC, University Medical Center Rotterdam, Rotterdam, Netherlands; 13 Division of Hematology, Department of Internal Medicine, Mayo Clinic, Rochester, MN USA; 14 Department of Immunology, Mayo Clinic, Rochester, USA; 15 Division of Hematology, Department of Medicine, Stanford University Medical Center, Stanford, CA USA; 16 Department of Hematology, Oncology Institute of Southern Switzerland and Institute of Oncology Research, Bellinzona, Switzerland; 17 Department of Oncology-Pathology, Science for Life Laboratory, Karolinska Institutet, Stockholm, Sweden; 18 Hematology Department and HCT Unit, G. Papanicolaou Hospital, Thessaloniki, Greece

**Keywords:** Cancer genetics, Chronic lymphocytic leukaemia

## Abstract

*SF3B1* mutations are recurrent in chronic lymphocytic leukemia (CLL), particularly enriched in clinically aggressive stereotyped subset #2. To investigate their impact, we conducted RNA-sequencing of 18 *SF3B1*^*MUT*^ and 17 *SF3B1*^*WT*^ subset #2 cases and identified 80 significant alternative splicing events (ASEs). Notable ASEs concerned exon inclusion in the non-canonical BAF (ncBAF) chromatin remodeling complex subunit, *BRD9*, and splice variants in eight additional ncBAF complex interactors. Long-read RNA-sequencing confirmed the presence of splice variants, and extended analysis of 139 CLL cases corroborated their association with *SF3B1* mutations. Overexpression of *SF3B1*^*K700E*^ induced exon inclusion in *BRD9*, resulting in a novel splice isoform with an alternative C-terminus. Protein interactome analysis of the BRD9 splice isoform revealed augmented ncBAF complex interaction, while exhibiting decreased binding of auxiliary proteins, including SPEN, BRCA2, and CHD9. Additionally, integrative multi-omics analysis identified a ncBAF complex-bound gene quartet on chromosome 1 with higher expression levels and more accessible chromatin in *SF3B1*^*MUT*^ CLL. Finally, Cancer Dependency Map analysis and BRD9 inhibition displayed BRD9 dependency and sensitivity in cell lines and primary CLL cells. In conclusion, spliceosome dysregulation caused by *SF3B1* mutations leads to multiple ASEs and an altered ncBAF complex interactome, highlighting a novel pathobiological mechanism in *SF3B1*^*MUT*^ CLL.

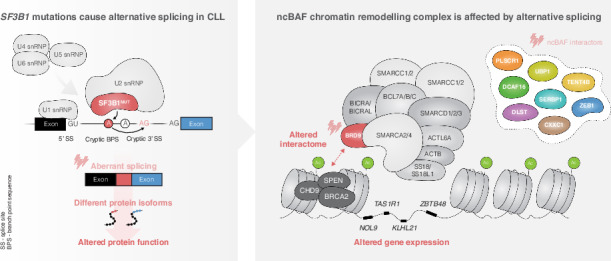

## Introduction

Pre-mRNA splicing is mediated by the spliceosome, a large ribonucleoprotein complex composed of five small nuclear ribonucleoproteins (snRNPs), each containing a small nuclear RNA (snRNA) – U1, U2, U4, U5, or U6. During splicing, the catalytic core component Splicing Factor 3b Subunit 1 (SF3B1) interacts with U1 and U2 to recognize 3′ and 5′ exon-intron boundary splice site motifs [1].

Recurrent *SF3B1* mutations have been reported in various cancer types, including chronic lymphocytic leukemia (CLL), and shown to induce altered splicing by alternative branch point usage or cryptic 3′ splice site selection [2–4]. *SF3B1* mutations in CLL, particularly within hotspot exons where the most frequent substitution is p.K700E [5], are associated with rapidly progressive disease [6, 7]. These mutations are enriched in patients belonging to stereotyped subset #2 (up to 45%) [8–10] and associated with poor response to chemoimmunotherapy and short overall survival [8, 11–14]. *SF3B1* mutations are often found at a subclonal level and expand during disease progression [15]. Accordingly, the occurrence of *SF3B1* mutations in early-stage CLL is about 5–10% and above 20% at advanced stages [5–7]. Mutated *SF3B1* in CLL has been associated with dysregulated DNA damage response, telomere dysfunction, increased Notch signaling mediated through a splice variant of *DVL2*, and activation of NF-κB signaling facilitated by alternative splicing of *MAP3K7* [3, 16]. In a mouse model, isolated *SF3B1* mutations led to cellular senescence, whereas mutated *SF3B1* in combination with *ATM* loss yielded a CLL-like disease in elderly mice [17].

Recently, *SF3B1* mutations in uveal melanoma (UVM), myelodysplastic syndrome (MDS), and CLL were associated with mis-splicing of Bromodomain Containing 9 (*BRD9*), a subunit of the non-canonical BRG1 (SMARCA4) or BRM (SMARCA2) associated factor (ncBAF) chromatin remodeling complex [18]. Functional studies in UVM indicated that correcting such mis-splicing may restore the tumor suppressor role of BRD9 [18]. The combinatorial assembly of BAF complexes, including canonical BAF (cBAF), polybromo-associated BAF (PBAF), and ncBAF varies between tissues, developmental stages, and disease states [19]. The ncBAF complex is defined by the presence of BRD9 and BICRA (also known as GLTSCR1) but shares common subunits with other BAF complex types [19].

We sought to further investigate *SF3B1* mutation-related alternative splicing events (ASEs) in CLL by performing RNA-sequencing (RNA-seq) in a cohort of 100 CLL cases carrying stereotyped B-cell receptors. Initially, we focused on subset #2 where we detected ASEs in several ncBAF complex-related transcripts, including BRD9. Extended analysis of other subset/non-subset cases and CLL cell lines confirmed our initial findings and led to the discovery of a novel BRD9 splice isoform with an alternative C-terminus, which could be induced by overexpression of mutated *SF3B1*. The alternative BRD9 isoform was shown to bind to the ncBAF complex and alter the interaction with auxiliary ncBAF complex proteins. Finally, drug response experiments revealed the sensitivity of both cell lines and primary CLL cells to a BRD9 inhibitor.

## Materials and methods

### Patient material

One hundred CLL cases carrying stereotyped B-cell receptors, including subsets #1 (n = 25), #2 (n = 35), #3 (n = 5), #4 (n = 14), #5 (n = 4), #6 (n = 4), #7 (n = 4), #59 (n = 1), #99 (n = 4), and #169 (n = 4), diagnosed according to the 2018 iwCLL criteria were evaluated [20]. In subset #2, there were 18 cases with *SF3B1* mutations (*SF3B1*^*MUT*^) and 17 cases without *SF3B1* mutations (*SF3B1*^*WT*^). Additionally, 7 more *SF3B1*^*MUT*^ cases were identified in the other subsets (Supplementary Table 1A). Informed consent was obtained according to the Declaration of Helsinki and each corresponding local Ethics Review Committee approved the study.

### RNA extraction, library construction, and short-read RNA-sequencing

Peripheral blood was collected and enriched for tumor cells (>95%) by FACS, bead-based sorting, or negative selection using the RosetteSep Human B Cell Enrichment Cocktail (Stemcell Technologies, Vancouver, BC, Canada). Sequencing libraries were constructed from 100 ng of high-quality RNA as assessed by Agilent 2100 Bioanalyzer (Agilent Technologies, Santa Clara, CA, USA) using the TruSeq Stranded Total RNA library preparation kit with ribosomal depletion through Ribo-Zero Gold treatment (Illumina, San Francisco, CA, USA). Libraries were multiplexed and sequenced on an Illumina HiSeq 2500 system using v4 sequencing chemistry and paired-end 125 base pair read length.

### Read mapping, processing, alternative splicing, and differential gene expression analyses

**Fig. 1:**
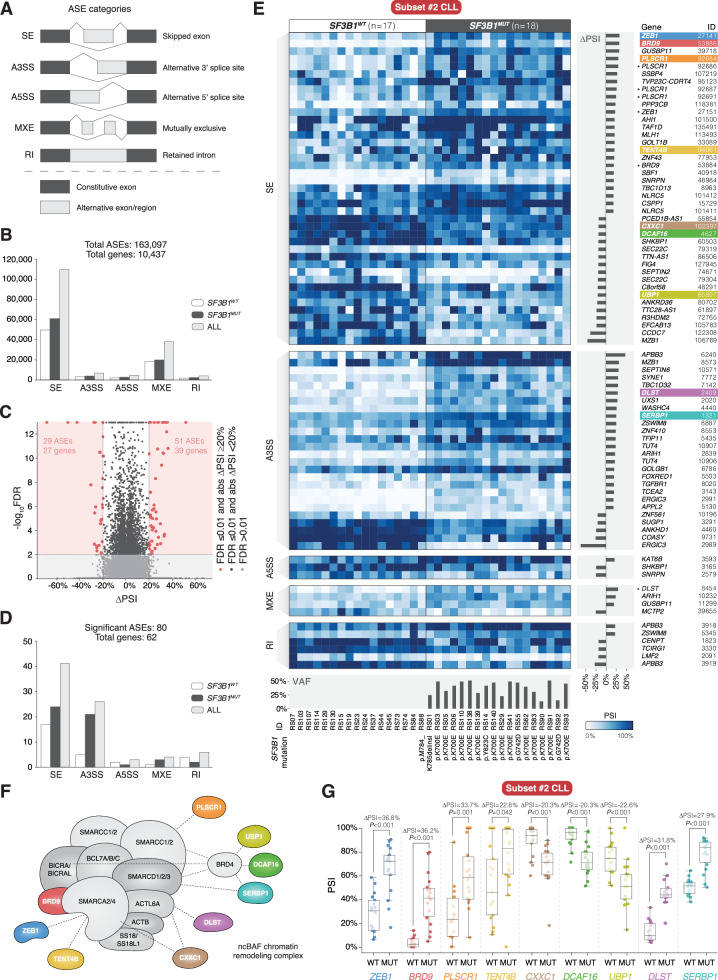
Alternative splicing analysis of *SF3B1*-mutated subset #2 CLL. **A** ASE categories, including skipped exon (SE), alternative 3′ splice site usage (A3SS), alternative 5′ splice site usage (A5SS), mutually exclusive exon usage (MXE), and intron retention (RI). **B** Bar plot displaying numbers of all identified ASEs in the comparison of 18 *SF3B1*^*MUT*^ and 17 *SF3B1*^*WT*^ subset #2 cases across different ASE categories. 163,097 ASEs were detected and involved transcripts of 10,437 genes. **C** Volcano plot depicting all identified ASEs in the comparison of *SF3B1*^*MUT*^ and *SF3B1*^*WT*^ subset #2 cases. Red dots indicate ASEs that are considered significant (|ΔPSI | ≥ 20% and FDR ≤ 0.01; 80 ASEs). **D** Bar plot displaying numbers of significant ASEs in the comparison of *SF3B1*^*MUT*^ and *SF3B1*^*WT*^ subset #2 cases across different ASE categories. 80 ASEs were considered significant and involved transcripts of 62 genes. **E** Heatmap illustrating the individual PSI values for the 80 significant ASEs detected in the comparison of *SF3B1*^*MUT*^ and *SF3B1*^*WT*^ subset #2 cases. 33 ASEs occurred within the same gene at least twice. ASEs are displayed based on the ASE category. The bar plot to the right of the heatmap shows ΔPSI values. The gene affected by each ASE and the corresponding unique ASE ID are listed. Genes that encode ncBAF complex-interacting proteins are depicted in color. For multiple ASEs per gene, only the top ASE is colored, while the others are marked with black asterisks. Additional details of these ASEs are provided in Supplementary Table 3A, B. **F** Model of the ncBAF chromatin remodeling complex and previously reported ncBAF complex interactors. Alternatively spliced transcripts identified in *SF3B1*^*MUT*^ subset #2 CLL that encode ncBAF complex-related proteins are depicted in color. **G** Scattered box plot showing the PSI value distribution, ΔPSI values, and *P* values (Wilcoxon rank-sum test) for significant ncBAF complex-related ASEs identified in the comparison of *SF3B1*^*MUT*^ and *SF3B1*^*WT*^ subset #2 cases. The specific ASEs for *ZEB1*, *BRD9*, *PLSCR1*, *TENT4B*, *CXXC1*, *DCAF16*, *UBP1*, *DLST*, and *SERBP1* have the unique ASE IDs ZEB1_SE_27141, BRD9_SE_53886, PLSCR1_SE_92684, PAPD5_SE_94061, CXXC1_SE_102397, DCAF16_SE_4627, UBP1_SE_85897, DLST_A3SS_2409, and SERBP1_A3SS_1321, respectively, in Supplementary Table 3A, B. The boxes represent the median and the interquartile range (IQR), while the whiskers extend to 1.5 times the IQR from the first and third quartiles. ASE: alternative splicing event; WT: wildtype; MUT: mutated; FDR: false discovery rate; PSI: percent spliced in; VAF: variant allele frequency.

FASTQ files were processed using the nf-core/rnaseq v1.0 pipeline with standard parameters [21], and the quality of short-read RNA-seq was assessed as detailed in Supplementary Information (Supplementary Table 2A, B). rMATS-turbo v4.1.2 was applied to categorize ASEs into the five types (Fig. 1A) and determine their exon inclusion rate referred to as percent spliced in (PSI) [22]. Only ASEs for which at least 80% of cases in a group had a measurable PSI value were retained. For each ASE, a ΔPSI value, i.e., the difference in average PSI values between groups, was calculated. ASEs with a false discovery rate (FDR) ≤ 0.01 and |ΔPSI | ≥ 20% were considered significant. DESeq2 v1.38.3 was used to normalize the count data and determine gene expression values [23].

### Long-read RNA-sequencing

Direct and PCR-based long-read RNA-seq was performed in the CLL cell line HG3 and CLL patient samples as detailed in Supplementary Information. The quality of the mapped files was examined using NanoComp v1.19.3 (Supplementary Table 2C) [24].

### Cell culture

The CLL cell lines MEC1, PCL12, HG3, PGA1, and CII, the AML cell line HNT34, the UVM cell line MEL202, and the HEK293T cell line were propagated as described in Supplementary Information.

### Inducible *SF3B1*^*K700E*^ and *SF3B1*^*WT*^ CLL cell lines

The CLL cell lines MEC1 and PCL12 were transduced by lentiviral vectors to overexpress *SF3B1*^*K700E*^ or *SF3B1*^*WT*^ under the control of a doxycycline-inducible promoter as detailed in Supplementary Information.

### PCR analysis of splice variants

Transcript levels were determined by qPCR using the PowerUp SYBR Green Master Mix (Applied Biosystems, Thermo Fisher Scientific, Waltham, MA, USA) on a CFX96 Touch Real-Time PCR Detection System (Bio-Rad Laboratories, Hercules, CA, USA) or by PCR using the REDTaq ReadyMix PCR Reaction Mix (Sigma-Aldrich, Merck, Darmstadt, Germany) on a SimpliAmp Thermal Cycler (Applied Biosystems) followed by agarose gel electrophoresis as described in Supplementary Information.

### Generation of stable cell lines overexpressing BRD9 splice isoforms

*BRD9* splice variants (Supplementary Information Appendix 1) were cloned from the cDNA of the MEC1 cell line and an *SF3B1*^*MUT*^ case into a lentiviral vector and stably overexpressed in the HEK293T cell line as detailed in Supplementary Information. Control cell lines were transduced with the same viral backbone as empty or overexpressing FLAG-V5-tagged yellow fluorescent protein (YFP).

### Co-immunoprecipitation and mass spectrometry analysis

HEK293T cell lines with stable overexpression of FLAG-V5-tagged YFP, the regular and alternative BRD9 isoforms were subjected to nuclear extraction, lysis, V5-tag-based co-immunoprecipitation, and subsequent analysis by mass spectrometry. Further information is provided in Supplementary Information.

### Analysis of publicly available data

RNA-seq and proteomic data from the Spanish CLL cohort (CLLE-ES; Supplementary Table 1B), generated within the International Cancer Genome Consortium (ICGC; http://platform.icgc-argo.org/), the Cancer Cell Line Encyclopedia (CCLE; https://sites.broadinstitute.org/ccle/), The Cancer Genome Atlas (TCGA; http://www.cbioportal.org/, Supplementary Table 1C and 7A, B), dbGaP (study accession phs001959.v1.p1), and ProteomeXchange (study accession PXD028936), were retrieved [25–34]. For analysis of BRD9 and histone chromatin binding, ChIP-sequencing (ChIP-seq) data was downloaded from the ENCODE database (https://www.encodeproject.org) for the chronic myeloid leukemia (CML) cell line K562 [35, 36]. Details are provided in Supplementary Information.

### Cancer Dependency Map and drug sensitivity database analyses

For the Cancer Dependency Map (DepMap) analysis, the shinyDepMap portal (https://labsyspharm.shinyapps.io/depmap) was queried with cluster size set to large, probability threshold set to 0, mixing ratio (shRNA:CRISPR) set to 20:80, and efficacy threshold set to 1 [37, 38]. The Genomics of Drug Sensitivity in Cancer (GDSC) database (https://www.cancerrxgene.org, Supplementary Table 9) was used to retrieve the sensitivity profile for I-BRD9 across 958 cell lines [39].

### Assessment of BRD9 drug sensitivity in CLL cell lines and primary CLL cells

Cell lines and primary CLL cells were treated with BRD9 inhibitors/degraders, I-BRD9 (SML1534; Sigma-Aldrich), PROTAC BRD9 Degrader-1 (HY-103632; MedChemExpress, New Jersey, NJ, USA), and dBRD9 Hydrochloride (SML2911; Sigma-Aldrich) [40, 41]. Dose-response analysis and assessment of proliferation and apoptosis were conducted as detailed in Supplementary Information.

### Statistics

Statistical methods, including tests for normality, variance, differences between groups, multiple testing correction, correlation, clustering, and dose-response curve fitting, were applied as described in Supplementary Information.

## Results

### *SF3B1*^*MUT*^ subset #2 cases display a distinct splicing signature linked to the ncBAF chromatin remodeling complex

Splicing analysis was performed in 18 *SF3B1*^*MUT*^ and 17 *SF3B1*^*WT*^ subset #2 cases (Supplementary Table 1A). Of all assessed ASEs (Fig. 1B, Supplementary Table 3A), 80 ASEs in transcripts of 62 genes were considered significant (Fig. 1C, Supplementary Table 3B; |ΔPSI | ≥ 20% and FDR ≤ 0.01) 51 (63.8%) and 29 (36.2%) of these had positive and negative ΔPSI values, respectively. Overall, *SF3B1*^*MUT*^ subset #2 cases displayed a higher proportion of positive ΔPSI values (Fig. 1D, Supplementary Fig. 1A) and the observed ASEs segregated *SF3B1*^*MUT*^ from *SF3B1*^*WT*^ subset #2 cases (Fig. 1E). When the analysis was expanded to encompass non-subset #2 subset cases (7 *SF3B1*^*MUT*^ and 58 *SF3B1*^*WT*^), including 39 cases from the poor-prognostic subset #1 and the favorable-prognostic subset #4 [42, 43], the splicing signature of *SF3B1*^*MUT*^ remained distinct compared to *SF3B1*^*WT*^ cases (Supplementary Fig. 1B). BRD9 emerged as particularly interesting as it is a subunit of the ncBAF chromatin remodeling complex [44]. In an extended protein-protein interaction network analysis using BioGRID v4.4 (https://thebiogrid.org), eight further ncBAF complex-interacting proteins were identified, ZEB1, PLSCR1, TENT4B (also known as PAPD5), CXXC1, DCAF16, UBP1, DLST, and SERBP1 (Fig. 1F, G, Supplementary Fig. 2A, B, Supplementary Table 4) [45].

### Long-read RNA-sequencing validates the alternatively spliced ncBAF complex-related transcripts

**Fig. 2:**
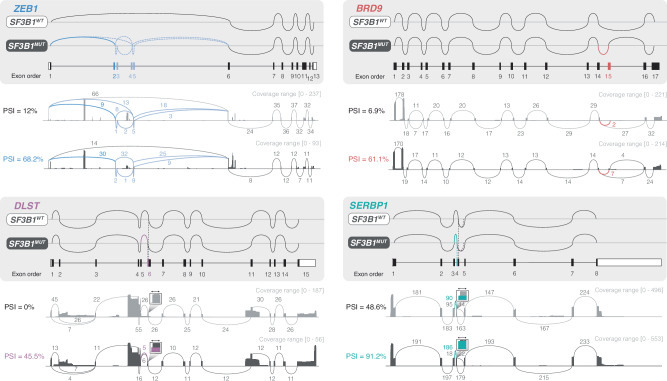
Long-read RNA-sequencing validates the predicted alternative transcripts related to the ncBAF complex in *SF3B1*-mutated CLL. Sashimi plots illustrating the identified ASEs and alternative splicing patterns in four genes that encode ncBAF complex-related proteins in *SF3B1*^*WT*^ versus *SF3B1*^*MUT*^ CLL; *ZEB1* and *BRD9* exhibit the top two ASEs in the SE category, while *DLST* and *SERBP1* showcase the top two ASEs in the A3SS category. Sashimi plots for other ncBAF complex-related genes, *PLSCR1*, *TENT4B*, *CXXC1*, *DCAF16*, and *UBP1*, are presented in Supplementary Fig. 3. For each gene, the top two sashimi plots within the gray box illustrate the predicted splice variants in *SF3B1*^*WT*^ versus *SF3B1*^*MUT*^ CLL. The colored arc highlights the primary ASE, while lighter arcs represent additional ASEs if present. The gene map indicates the relative location and order of the detected exons in relation to the sequencing results. For each corresponding gene, the lower two sashimi plots show the coverage and splice junction count data from the aligned long-read RNA-seq data from an *SF3B1*^*WT*^ case (RS24) and an *SF3B1*^*MUT*^ case (RS55), both belonging to subset #2 CLL. The direction of the genes is arranged from left to right. WT: wildtype; MUT: mutated; PSI: percent spliced in.

Long-read RNA-seq confirmed the presence of the full-length alternatively spliced ncBAF complex-related transcripts (Fig. 2, Supplementary Fig. 3). We also re-analyzed publicly available long-read RNA-seq data from CLL, including cases published by Tang et al., using FLAIR [26]. Overlapping ASEs, including the ASE in *SERBP1*, were identified in *SF3B1*^*MUT*^ cases, as in our analyses of short-read RNA-seq data (Supplementary Fig. 4A, B, Supplementary Table 5).

### Extended analyses of ncBAF complex-related alternative splicing events

We investigated the observed ncBAF complex-related ASEs in the remaining 65 subset cases (Supplementary Table 1A and 3C, D) and identified similar splicing differences in *SF3B1*^*MUT*^ versus *SF3B1*^*WT*^ for *ZEB1*, *BRD9*, *DCAF16*, *DLST*, and *SERBP1* (Supplementary Fig. 1C). Subsequent analysis of RNA-seq data from ICGC CLLE-ES [30, 31], including 8 *SF3B1*^*MUT*^ and 66 *SF3B1*^*WT*^ cases (Supplementary Table 1B), identified 85 significant ASEs in transcripts of 71 genes (Supplementary Fig. 5A–G, Supplementary Table 6A, B). A significant overlap was observed between the genes targeted by *SF3B1*^*MUT*^-related ASEs in the ICGC CLLE-ES and subset #2 cases, including *BRD9*, *DLST*, and *SERBP1*. Furthermore, we analyzed a set of 1019 cancer cell lines from CCLE [28], including 4 *SF3B1*^*MUT*^ cell lines, and detected significant exon usage differences within six of the ncBAF complex-related ASEs identified in the comparison of *SF3B1*^*MUT*^ and *SF3B1*^*WT*^ subset #2 cases (Supplementary Fig. 6). Finally, we noted a strong correlation between the RNA-seq-based *SF3B1*^*MUT*^ variant allele frequency (VAF) and the PSI values for  most of the ncBAF complex-related ASEs, both in our subset cases and the ICGC CLLE-ES cases (Supplementary Fig. 7A, B) [30, 31].

### Alternative *BRD9* splicing in CLL and other cancer types

The most notable ASE in our analysis concerned exon 15 inclusion in *BRD9*. Consistent with our findings, the analysis of long-read RNA-seq data from Tang et al. [26] showed high exon 15 inclusion in *BRD9* in an *SF3B1*^*MUT*^ case (PSI of 45.1%) contrasting with low exon inclusion in an *SF3B1*^*WT*^ case (PSI of 2.6%) (Supplementary Fig. 4C).

Since exon 15 inclusion in *BRD9* has been reported to lead to nonsense-mediated decay and lower transcript levels in UVM and MDS, we sought to investigate *BRD9* gene expression levels in additional cancer types that harbor *SF3B1* mutations [18]. In a re-analysis of the UVM cases from TCGA [27, 29, 33, 34], a significant 0.45-fold gene expression difference was found in *SF3B1*^*MUT*^ compared to *SF3B1*^*WT*^ cases (Wilcoxon rank-sum test, *P* < 0.001); however, no significant differences were seen in other cancer types (Supplementary Fig. 8, Supplementary Table 7A, B). Furthermore, no significant differences in *BRD9* gene/protein expression between *SF3B1*^*MUT*^ and *SF3B1*^*WT*^ cases were identified in three independent gene expression and one proteomic dataset from CLL (Supplementary Fig. 9A–J).

### *SF3B1* mutations induce alternative *BRD9* splicing and the formation of a splice isoform with an alternative C-terminus

**Fig. 3:**
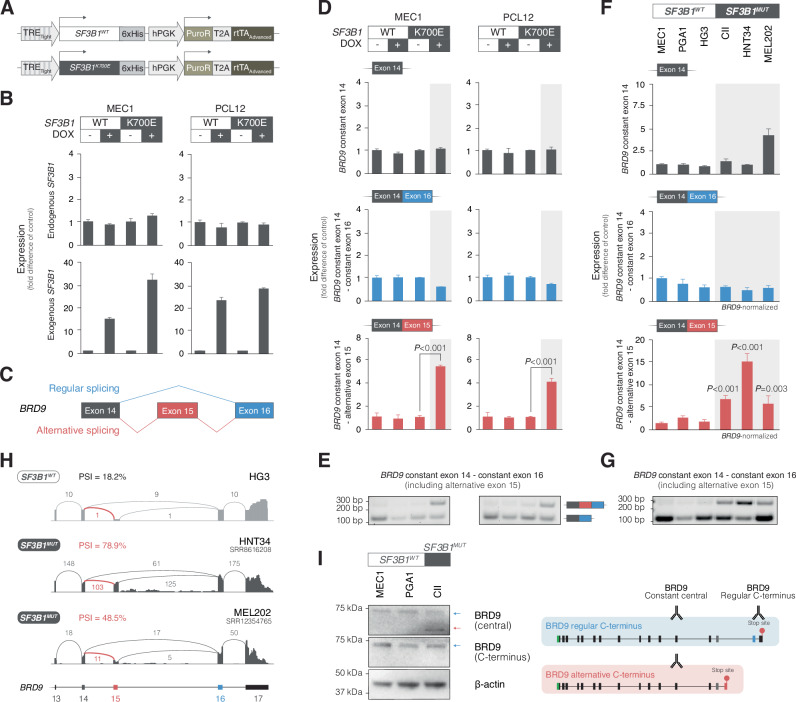
*SF3B1* mutations induce alternative splicing of *BRD9* resulting in a splice isoform with an alternative C-terminus. **A** Scheme illustrating lentiviral constructs for *SF3B1*^*WT*^ and *SF3B1*^*K700E*^ overexpression under the control of a DOX-inducible promoter. **B** Bar plots depicting expression levels of endogenous and exogenous *SF3B1* upon overexpression of *SF3B1*^*WT*^ and *SF3B1*^*K700E*^ in MEC1 and PCL12 cell lines. The bar plots display the mean values of triplicates with error bars representing the standard deviation. **C** Scheme illustrating the identified ASE in *BRD9* with the regular and alternative splicing pattern. The blue and red boxes illustrate constant and alternative exons, respectively. **D** Increased expression of the alternative *BRD9* transcript upon overexpression of *SF3B1*^*K700E*^ as compared to *SF3B1*^*WT*^ in MEC1 and PCL12 cell lines. The checkerboard scheme at the top of the panel illustrates the experimental conditions. The bar plots display the expression levels of *BRD9* transcripts in general (transcripts containing exon 14), the regular *BRD9* transcript (transcripts containing exon 14 followed by exon 16), and the alternative *BRD9* transcript (transcripts containing exon 14 followed by exon 15) as determined by qPCR. Results are normalized on *GAPDH* levels and expressed as fold difference between DOX+ and DOX− conditions with corresponding *P* values (Student’s *t*-test). The bar plots display the mean values of experimental triplicates, with error bars representing the standard deviation. **E** Agarose gel electrophoresis images of PCR products spanning from exon 14 to 16 and allowing for exon 15 inclusion show a higher abundance of alternative *BRD9* transcript in *SF3B1*^*K700E*^ DOX+. For a complete gel image see Supplementary Fig. 13A. **F** Assessment of *BRD9* splice variants in *SF3B1*^*WT*^ and *SF3B1*^*MUT*^ CLL, AML, and UVM cell lines. MEC1, PGA1, and HG3 are *SF3B1*^*WT*^ CLL cell lines, CII an *SF3B1*^*K666E*^ CLL cell line, HNT34 an *SF3B1*^*K700E*^ AML cell line, and MEL202 an *SF3B1*^*R625G*^ UVM cell line. The bar plots display the expression levels of *BRD9* transcripts in general, the regular *BRD9* transcript, and the alternative *BRD9* transcript as determined by qPCR. Results for *BRD9* transcripts in general are normalized on *GAPDH* levels, whereas regular and alternative *BRD9* transcript levels are normalized on levels of *BRD9* transcripts in general and expressed as fold difference between MEC1 and other cell lines with corresponding *P* values (Student’s *t*-test). The bar plots display the mean values of experimental triplicates, with error bars representing the standard deviation. **G** Agarose gel electrophoresis image of PCR products spanning from exon 14 to 16 and allowing for exon 15 inclusion shows a higher abundance of alternative *BRD9* transcript in *SF3B1*^*MUT*^ cell lines. For a complete gel image see Supplementary Fig. 13A. **H** Sashimi plots from the *SF3B1*^*WT*^ CLL cell line HG3 and the *SF3B1*^*MUT*^ AML and UVM cell lines, HNT34 and MEL202, respectively, illustrating the alternatively spliced exon 15 in *BRD9*. The plots for HG3 are based on direct long-read RNA-seq, and HNT34 (SRR8616208) and MEL202 (SRR12354765) on short-read RNA-seq. **I** BRD9 splice isoform expression in *SF3B1*^*WT*^ and *SF3B1*^*MUT*^ CLL cell lines determined by isoform-specific antibodies. An antibody specific to the C-terminus of the regular BRD9 isoform detected a single band in both *SF3B1*^*WT*^ and *SF3B1*^*MUT*^ cell lines. Conversely, an antibody targeting a constant epitope within the central part of BRD9, detected two bands in the *SF3B1*^*MUT*^ cell line CII and one band in the *SF3B1*^*WT*^ cell lines MEC1 and PGA1, corresponding to the regular higher molecular weight BRD9 isoform and the alternative lower molecular weight BRD9 isoform. For complete blot images see Supplementary Fig. 13A. WT: wildtype; MUT: mutated; DOX: doxycycline.

To explore the role of *SF3B1* mutations in alternative splicing of *BRD9*, we used inducible *SF3B1*^*K700E*^ and *SF3B1*^*WT*^ overexpression in the CLL cell lines MEC1 and PCL12 (Fig. 3A–C). Overexpression of *SF3B1*^*K700E*^ selectively increased the levels of the alternative *BRD9* splice variant (Fig. 3D, E; MEC1 and PCL12, Student’s *t*-test, *P* < 0.001 and *P* < 0.001, respectively). The expression of the regular and alternative *BRD9* splice variants was also analyzed in 3 *SF3B1*^*WT*^ cell lines, MEC1, PGA1, and HG3 (all CLL), and 3 *SF3B1*^*MUT*^ cell lines, CII (CLL), HNT34 (AML), and MEL202 (UVM). *SF3B1*^*MUT*^ cell lines demonstrated significantly higher expression levels of the alternative *BRD9* splice variant compared to *SF3B1*^*WT*^ cell lines (Fig. 3F, G). Moreover, analysis of short-read RNA-seq data from 2 *SF3B1*^*MUT*^ cell lines, HNT34 and MEL202, demonstrated high exon 15 inclusion (PSI of 78.9% and 48.5%, respectively) (Fig. 3H).

Based on the splicing pattern observed in *BRD9*, we anticipated that the alternative splicing yields a BRD9 isoform with an alternative C-terminus. Using anti-BRD9 antibodies against different epitopes, we noted that the *SF3B1*^*MUT*^ CII cell line expressed an isoform shorter than the regular 67 kDa isoform that could only be detected with an antibody specific for the central part of the protein, but not with an antibody specific for the C-terminus of the regular BRD9 isoform (Fig. 3I). In contrast, the *SF3B1*^*WT*^ cell lines showed a single 67 kDa band of the regular BRD9 isoform that could be detected by both antibodies.

### Cloning of *BRD9* splice variants and interactome analysis

**Fig. 4:**
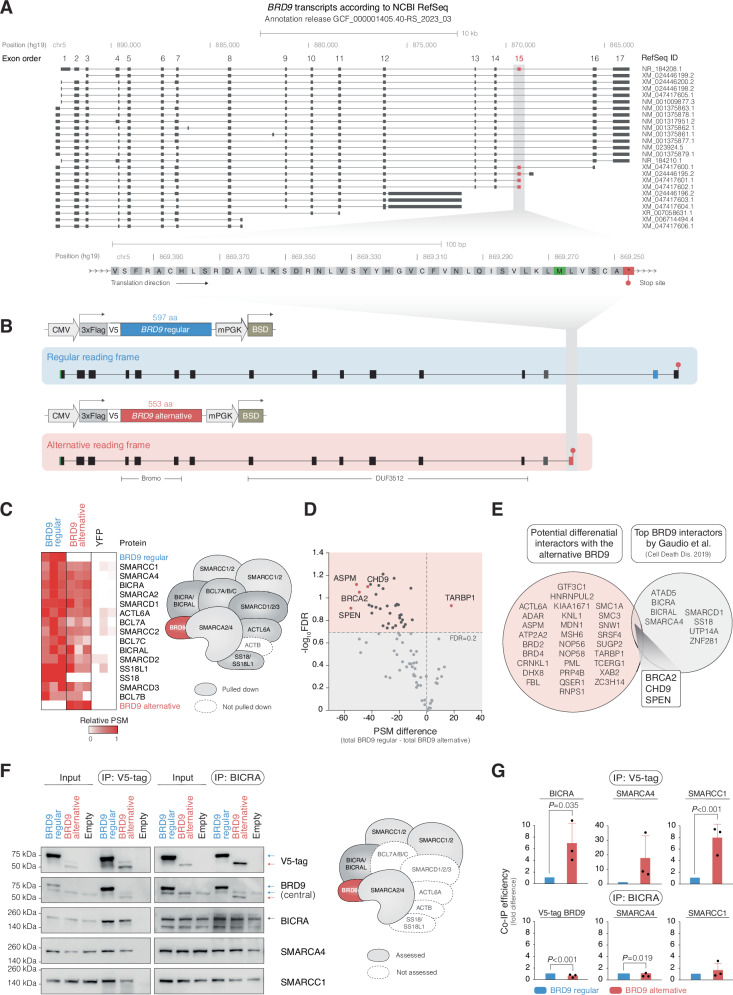
The regular and alternative BRD9 isoforms display different interactomes. **A** Scheme illustrating *BRD9* splice variants and predicted protein isoforms according to NCBI RefSeq (Annotation release GCF_000001405.40-RS_2023_03). The zoom-in view highlights the predicted protein sequence stemming from exon 15 inclusion in *BRD9*. Upon translation of exon 15, a stop codon emerges near the end of the exon, resulting in a shorter splice isoform with an alternative C-terminus. **B** Scheme depicting lentiviral constructs for overexpression of the regular and alternative *BRD9* splice variants. The corresponding DNA and protein sequences are available in Supplementary Information Appendix 1. **C** Heatmap illustrating the detected ncBAF complex subunits upon co-immunoprecipitation and subsequent mass spectrometry analysis with the regular or alternative BRD9 isoforms stably overexpressed in the HEK293T cell line. The relative protein levels are based on the total number of identified peptide-spectrum matches (PSMs) for the corresponding protein from the mass spectrometry analysis. The ncBAF complex model to the right of the heatmap illustrates that the alternative BRD9 isoform precipitated a majority of the ncBAF complex subunits. ACTB was not detected among the co-immunoprecipitates. The control cells expressing FLAG-V5-tagged YFP were used. **D** Volcano plot displaying differential interaction analysis of proteins that selectively interacted more with the regular or alternative BRD9 isoforms (multiple Student’s *t*-tests with Benjamini–Hochberg multiple testing correction). The top 5 candidates are highlighted in red. **E** Venn diagram showing a comparison of the significant differentially interacting proteins with the regular or alternative BRD9 isoforms and BRD9-interacting proteins reported by Gaudio et al. [46]. The intersecting proteins are SPEN, BRCA2, and CHD9. **F** Western blot analysis of V5-tag and BICRA co-immunoprecipitates in the HEK293T cell line with stable overexpression of the regular and alternative BRD9 isoforms. In the V5-tag-immunoprecipitations, both overexpressed BRD9 isoforms precipitated BICRA together with SMARCA4 and SMARCC1. Reciprocal BICRA co-immunoprecipitations precipitated both overexpressed BRD9 isoforms, SMARCA4 and SMARCC1. The control cells were transduced with an empty lentiviral vector. For complete blot images see Supplementary Fig. 13A. **G** Bar plots showing V5-tag and BICRA co-immunoprecipitation efficiency with BRD9, BICRA, SMARCA4, and SMARCC1. Results are expressed as fold differences between the regular and alternative BRD9 isoforms with corresponding *P* values (Student’s *t*-test). The bar plots display the mean values from three repeated experiments, with error bars representing the standard deviation. For detailed quantification and calculation see Supplementary Fig. 13B. PSM: peptide-spectrum matches; FDR: false discovery rate; IP: immunoprecipitation.

According to NCBI RefSeq, the inclusion of exon 15 in *BRD9* is predicted to add an alternative C-terminus to *BRD9*, where the regular 89 amino acids in the C-terminus encoded by exons 16 and 17 are exchanged for 45 amino acids encoded by exon 15 (Fig. 4A).

The two *BRD9* splice variants were cloned and overexpressed in the HEK293T cell line by lentiviral transduction (Fig. 4B). Subsequently, their interactomes were investigated by co-immunoprecipitation and mass spectrometry. Both splice isoforms bound to most ncBAF complex subunits (Fig. 4C, Supplementary Table 8A–C). The alternative BRD9 isoform showed a significantly lower interaction with 33 proteins, including SPEN, BRCA2, ASPM, and CHD9, and a higher interaction with TARBP1 (Fig. 4D). In comparison with 11 previously reported BRD9 interactors, SPEN, BRCA2, and CHD9 intersected with these auxiliary ncBAF complex proteins (Fig. 4E) [46].

The interaction with BICRA, SMARCA4, and SMARCC1 was recapitulated in independent co-immunoprecipitation experiments (Fig. 4F). In addition, the alternative BRD9 isoform displayed a higher binding capacity for these ncBAF complex subunits as compared to the regular BRD9 isoform (Fig. 4G).

### A gene quartet on chromosome 1 bound by *BRD9* is highly expressed in *SF3B1*^*MUT*^ CLL

**Fig. 5:**
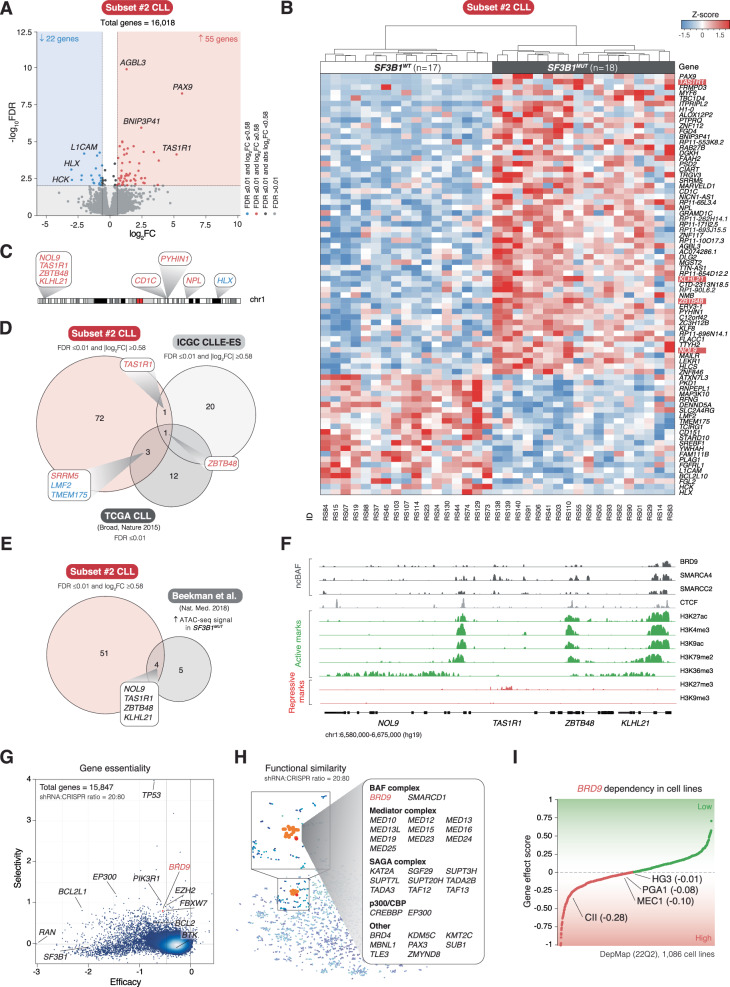
*BRD9* binds a gene quartet on chromosome 1 and displays selective dependency in DepMap. **A** Volcano plot depicting differentially expressed genes between 18 *SF3B1*^*MUT*^ and 17 *SF3B1*^*WT*^ subset #2 cases with 22 downregulated and 55 upregulated genes ( | log_2_FC | ≥ 0.58 and FDR < 0.01). **B** Unsupervised clustermap illustrating the distinct clustering of *SF3B1*^*MUT*^ and *SF3B1*^*WT*^ subset #2 cases based on differential gene expression. The four neighboring genes, *NOL9*, *TAS1R1*, *ZBTB48*, and *KLHL21*, located on chromosome 1 are depicted in color. The Ward method and the Euclidean metric were employed. **C** Karyoplot of chromosome 1 displaying differentially expressed genes between *SF3B1*^*MUT*^ and *SF3B1*^*WT*^ subset #2 cases. Examination of chromosomal positions revealed four neighboring genes, *NOL9*, *TAS1R1*, *ZBTB48*, and *KLHL21*. **D** Venn diagram showing the overlap of genes with differential expression between *SF3B1*^*MUT*^ and *SF3B1*^*WT*^ cases in three independent datasets; subset #2 CLL, ICGC CLLE-ES [30, 31], and TCGA CLL [29, 33, 34]. For the differential gene expression analyses of the latter two datasets see Supplementary Fig. 10A–E. *ZBTB48* appeared upregulated in all three datasets, while *TAS1R1* in subset #2 CLL and ICGC CLLE-ES. **E** Venn diagram showing the overlap of genes with higher expression levels in *SF3B1*^*MUT*^ subset #2 cases and increased chromatin accessibility in *SF3B1*^*MUT*^ CLL cases from Beekman et al. [47]. The overlap comprises *NOL9*, *TAS1R1*, *ZBTB48*, and *KLHL21*. **F** Coverage tracks from ChIP-seq data sourced from the ENCODE database [35, 36], based on the CML cell line K562, illustrating the binding of BRD9 and the ncBAF complex subunits SMARCA4 and SMARCC2 to the gene quartet region on chromosome 1. The promoters for these genes appear active based on the signal for H3K27ac, H3K4me3, H3K9ac, and H3K79me2, and transcriptionally active based on the H3K36me3 signal, whereas transcriptional repression is low based on the low signal for H3K27me3 and H3K9me3. **G** Gene essentiality map showing the relationship between efficacy and selectivity of genes in shinyDepMap [37, 38]. *SF3B1* appears as a non-selective dependency with high efficacy, while *BRD9* as a selective dependency with high efficacy. *BRD9* is in the dependency region of *PIK3R1*, *EZH2*, and *FBXW7*. **H** Functional similarity clustering showing a chromatin remodeling dependency cluster connected to the *BRD9* dependency signature in shinyDepMap. *BRD9* clusters with *SMARCD1*, a BAF complex subunit, subunits of the Mediator complex, p300/CBP subunits, and subunits of the SAGA complex. **I** *BRD9* dependency ranking of the 1086 DepMap (22Q2) cell lines. For CLL cell lines, gene effect scores are given within the parenthesis. WT: wildtype; MUT: mutated; FDR: false discovery rate; FC: fold change.

Comparison between *SF3B1*^*MUT*^ and *SF3B1*^*WT*^ subset #2 cases revealed 55 and 22 significantly higher and lower expressed genes in *SF3B1*^*MUT*^ cases, respectively (Fig. 5A, B; |log_2_FC | ≥ 0.58 and FDR < 0.01). Analysis of the chromosomal positions of differentially expressed genes identified four adjacent genes on chromosome 1, *NOL9*, *TAS1R1*, *ZBTB48*, and *KLHL21*, which were upregulated in *SF3B1*^*MUT*^ subset #2 cases (Fig. 5C). In a similar comparison of RNA-seq data from ICGC CLLE-ES [30, 31] (Supplementary Fig. 10A, B) and TCGA CLL [29, 33, 34] (Supplementary Fig. 10C–E), we observed an overlap between the 3 datasets, indicating upregulation of *ZBTB48* and *TAS1R1* in *SF3B1*^*MUT*^ (Fig. 5D). By comparing the differentially expressed genes in *SF3B1*^*MUT*^ subset #2 cases with published ATAC-sequencing data of *SF3B1*^*MUT*^ CLL [47], the gene quartet also exhibited increased chromatin accessibility in *SF3B1*^*MUT*^ CLL (Fig. 5E). ChIP-seq data of the CML cell line K562 indicated that BRD9, SMARCA4, and SMARCC2 can bind to the gene quartet (Fig. 5F). Furthermore, based on the high signal for H3K27ac, H3K4me3, H3K9ac, H3K79me2, and H3K36me3, and the low signal for H3K27me3 and H3K9me3, these genes appear transcriptionally active [35, 36].

### *BRD9* displays a selective dependency

In a DepMap analysis, which assesses gene dependency based on CRISPR and RNA interference data in cancer cell lines [37, 38], *BRD9* appeared to have relatively high selectivity and efficacy (Fig. 5G). In detail, *BRD9* was located together with other known cancer dependencies, including *PIK3R1*, *EZH2*, and *FBXW7*, whereas *SF3B1* had a very high efficacy but low selectivity. Upon functional similarity clustering, *BRD9* clustered with the BAF complex subunit *SMARCD1* and multiple genes from the transcriptional regulator Mediator complex and the Spt-Ada-Gcn5 Acetyltransferase (SAGA) complex (Fig. 5H). Of the included CLL cell lines, the *SF3B1*^*MUT*^ CII cell line displayed a higher dependency on *BRD9* than the 3 *SF3B1*^*WT*^ cell lines MEC1, PGA1, and HG3 (Fig. 5I).

### *BRD9* inhibition reveals cytotoxic effects in cell lines and primary CLL cells

**Fig. 6:**
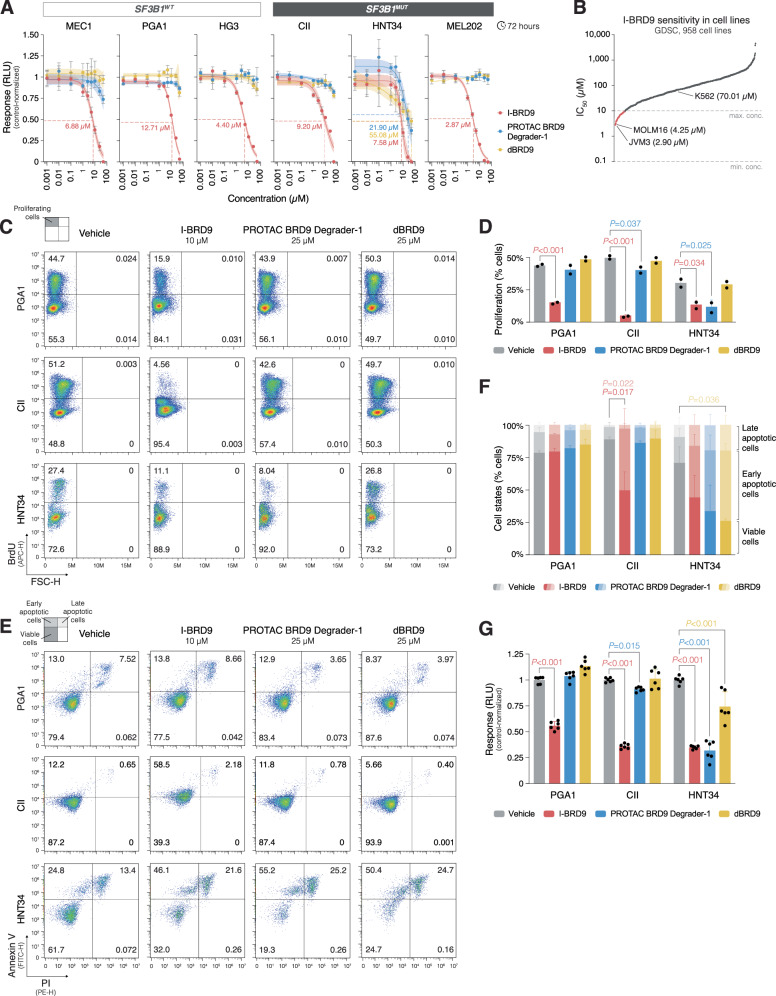
*BRD9* inhibition exhibits anti-proliferative and pro-apoptotic effects in *SF3B1*-mutated cell lines. **A** Dose-response analysis of I-BRD9, PROTAC BRD9 Degrader-1, and dBRD9 treatments in 3 *SF3B1*^*WT*^ cell lines, MEC1, PGA1, and HG3 (all CLL), and 3 *SF3B1*^*MUT*^ cell lines, CII (CLL), HNT34 (AML), and MEL202 (UVM). The cell lines were treated with drug concentrations ranging from 0.001 to 50 µM for 3 days, and cell viability was determined by CellTiter-Glo 2.0. Complete cell killing was exclusively observed with I-BRD9 treatment, allowing for the determination of corresponding IC_50_ values for each cell line. The HNT34 cell line exhibited sensitivity to all three drugs, thereby allowing for the determination of IC_50_ values for all conditions. Dose-response curves are shown with 95% confidence intervals, while individual dots display the mean values of triplicates, with error bars representing the standard deviation. **B** I-BRD9 sensitivity profile in 958 cancer cell lines from GDSC [39]. **C** Assessment of proliferation in an *SF3B1*^*WT*^ cell line, PGA1, and *SF3B1*^*MUT*^ cell lines, CII and HNT34, upon treatment with 10 µM I-BRD9, 25 µM PROTAC BRD9 Degrader-1, or 25 µM dBRD9. Cell lines were treated for 3 days with a 5-hour exposure to BrdU at the end, and proliferation was quantified by flow cytometry, measured as BrdU+ cells. Vehicle (DMSO) was used as the negative control for drug treatment, while cells not exposed to BrdU served as the negative control to assess the specificity of the anti-BrdU antibody. The upper left quadrants (BrdU+ cells) in the density plots represent the percentages of proliferating cells. **D** Bar plot displaying differences in the percentages of proliferating cells compared to negative controls with corresponding *P* values (one-way ANOVA). The bar plot displays the mean values from two repeated experiments, with error bars representing the standard deviation. **E** Assessment of apoptosis in the same cell lines and the identical samples as in (**C**). Treatment with 5 µM Camptothecin and vehicle (DMSO) were used as the positive and negative controls, respectively. Apoptosis was evaluated by Annexin V/PI staining and subsequent flow cytometry analysis. The lower left (Annexin V-/PI- cells), upper left (Annexin V+/PI- cells), and upper right (Annexin V+/PI+ cells) quadrants in the density plots represent the percentages of viable, early apoptotic, and late apoptotic cells, respectively. **F** Stacked bar plot displaying differences in the percentages of viable, early apoptotic, and late apoptotic cells compared to negative controls with corresponding *P* values (one-way ANOVA). The bar plot displays the mean values from two repeated experiments, with error bars representing the standard deviation. **G** Bar plot showing cell viability differences as determined by CellTiter-Glo 2.0 in the same cell lines under the same experimental conditions as in panels (**C**, **E**) with corresponding *P* values (one-way ANOVA). The bar plot displays the mean values from two repeated experiments in triplicates, with error bars representing the standard deviation. WT: wildtype; MUT: mutated; IC_50_: half-maximal inhibitory concentration; RLU: relative luminescence unit.

The differential sensitivity of *SF3B1*^*MUT*^ and *SF3B1*^*WT*^ cell lines towards chemical BRD9 inhibition was evaluated through exposure to I-BRD9, PROTAC BRD9 Degrader-1, and dBRD9. Complete cell killing was achieved exclusively with I-BRD9 treatment, allowing for the determination of the corresponding half-maximal inhibitory concentration (IC_50_) values for each cell line (Fig. 6A). The cell lines demonstrated similar sensitivity to I-BRD9 treatment with no apparent response to PROTAC BRD9 Degrader-1 and dBRD9 treatment, except for the *SF3B1*^*MUT*^ AML HNT34 cell line, which showed sensitivity to all three drugs. Similarly, HEK293T cell lines stably overexpressing regular and alternative BRD9 isoforms exhibited sensitivity only to I-BRD9, with no discernible difference in response between the two conditions (Supplementary Fig. 11).

In a GDSC query assessing I-BRD9 sensitivity across 958 cell lines, the cell lines susceptible to BRD9 inhibition were predominantly from hematological malignancies (89% compared to the expected 17%), notably including CLL (Fig. 6B, Supplementary Table 9) [39].

The *SF3B1*^*WT*^ cell line PGA1 and the *SF3B1*^*MUT*^ cell lines CII and HNT34 were further assessed for anti-proliferative and pro-apoptotic effects upon treatment with 10 µM I-BRD9, 25 µM PROTAC BRD9 Degrader-1, and 25 µM dBRD9 through BrdU incorporation (Fig. 6C, D) and Annexin V binding (Fig. 6E, F). The most potent anti-proliferative and pro-apoptotic effects were observed with I-BRD9, particularly in the CII cell line (44.9% decrease in proliferating cells, and 38.0% increase in early apoptotic cells compared to control, one-way ANOVA, *P* < 0.001 and *P* = 0.017, respectively). Treatment with PROTAC BRD9 Degrader-1 yielded pro-apoptotic properties primarily in the HNT34 cell line, while also demonstrating some anti-proliferative effects in the CII and HNT34 cell lines. Apoptosis induction upon treatment with dBRD9 was observed exclusively in the HNT34 cell line, with no apparent anti-proliferative effects detected across any of the tested cell lines. Comparable results were obtained using cell viability measurements on the same set of cells (Fig. 6G).

**Fig. 7:**
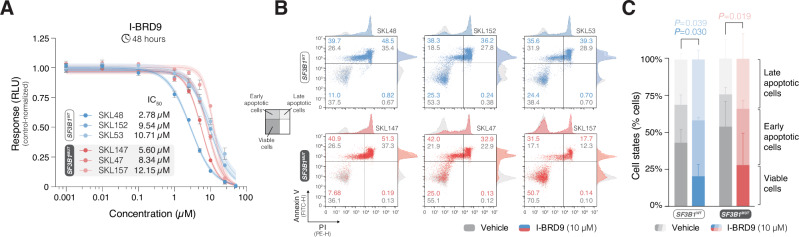
BRD9 inhibition induces potent pro-apoptotic effects in primary CLL cells. **A** Dose-response analysis of I-BRD9 treatment in 3 *SF3B1*^*WT*^ (SKL48, SKL152, SKL53) and 3 *SF3B1*^*MUT*^ (SKL147, SKL47, SKL157) primary CLL cell samples. The cell lines were treated with drug concentrations ranging from 0.001 to 50 µM for 2 days, and cell viability was determined by CellTiter-Glo 2.0. Dose-response curves are shown with 95% confidence intervals, while individual dots display the mean values of triplicates, with error bars representing the standard deviation. **B** Stacked density plots showing apoptosis assessment in the same primary CLL cell samples as in (**A**). Treatment with 5 µM Camptothecin and vehicle (DMSO) were used as the positive and negative controls, respectively. Apoptosis was evaluated by Annexin V/PI staining and subsequent flow cytometry analysis. The lower left (Annexin V-/PI- cells), upper left (Annexin V+/PI- cells), and upper right (Annexin V+/PI+ cells) quadrants in the density plots represent the percentages of viable, early apoptotic, and late apoptotic cells, respectively. **C** Stacked bar plot displaying differences in the percentages of viable, early apoptotic, and late apoptotic cells compared to negative controls with corresponding *P* values (Student’s *t*-test). The bar plot displays the mean values, with error bars representing the standard deviation. WT: wildtype; MUT: mutated; IC_50_: half-maximal inhibitory concentration; RLU: relative luminescence unit.

Sensitivity to BRD9 inhibition was also assessed in primary CLL cells. Treatment with I-BRD9 showed similar IC_50_ values between *SF3B1*^*MUT*^ and *SF3B1*^*WT*^ cells (Fig. 7A; mean IC_50_ of 8.7 µM and 7.7 µM, respectively), while no response to PROTAC BRD9 Degrader-1 and dBRD9 treatment was observed (Supplementary Fig. 12A, B). Furthermore, the same primary CLL cells were examined for induced apoptosis at the same drug concentrations as for cell lines. I-BRD9 induced comparable pro-apoptotic effects between *SF3B1*^*MUT*^ and *SF3B1*^*WT*^ cells (Fig. 7B, C; 16.3% and 12.3% of early apoptotic cells compared to control, Student’s *t*-test, *P* = 0.019 and *P* = 0.039, respectively), whereas PROTAC BRD9 Degrader-1 and dBRD9 did not induce apoptosis (Supplementary Fig. 12C, D).

## Discussion

*SF3B1* mutations and spliceosome dysregulation have been associated with a particularly dismal outcome in CLL [3, 5–7, 10, 18, 48]. Here, we report that a prominent feature in *SF3B1*^*MUT*^ CLL concerns the inclusion of an alternative exon in the ncBAF chromatin remodeling complex subunit *BRD9*, resulting in a novel splice isoform with an alternative C-terminus [49]. Additional ASEs were observed in transcripts encoding for proteins also reported to interact with the ncBAF complex.

The strength of the study lies in the initial study cohort, which consisted of a uniform patient subgroup, specifically subset #2 CLL. Our analysis identified a greater number of SE ASEs than previous studies [3, 50], potentially due to differences in study cohorts and the bioinformatics tools applied; relevant to mention in that regard, we utilized contemporary, actively maintained tools to identify ASEs. The observed ASEs occurred to varying degrees in *SF3B1*^*MUT*^ cases, likely due to the heterozygous state of *SF3B1* mutations, allowing both normal and perturbed splicing. This may also stem from varying VAFs, where different admixtures of *SF3B1*^*MUT*^ cells exist within the cancer cell population [15]. Consistent with this, we observed a strong correlation between PSI values and *SF3B1* VAF.

The potential significance of the multiple identified ASEs in transcripts encoding ncBAF complex-related proteins was examined using nanopore-based long-read RNA-seq, which confirmed many of the ASEs identified in our short-read RNA-seq analysis. Furthermore, they were observed in extended analyses of independent cohorts, showing that aberrant splicing extends beyond subset #2 CLL. On these grounds, the ncBAF complex may be targeted at multiple levels in *SF3B1*^*MUT*^ CLL beyond *BRD9*. In addition to these alterations, BAF complex subunits *SMARCC1* and *ARID1A* are deleted or mutated in 2% of CLL cases each [51].

The role of BRD9 in oncogenesis appears two-sided since BRD9 has been shown to either drive or suppress cancer phenotypes [18, 52]. The inclusion of exon 15 in *BRD9* occurs in proximity to the DUF3512 domain which is critical for the integrity of the ncBAF complex [18, 52]. In UVM, alternative splicing of *BRD9* in connection to *SF3B1* mutations was reported to decrease BRD9 protein levels due to nonsense-mediated decay [18]. Here, we also established a causative connection between mutated *SF3B1* and alternative splicing of *BRD9*. However, in contrast to UVM, we did not observe notably lower gene/protein expression levels in CLL. Instead, we detected a novel shorter BRD9 splice isoform with an alternative C-terminus caused by the exon 15 inclusion.

In the BRD9 interactome analysis, both BRD9 splice isoforms were found to bind to most subunits of the ncBAF complex, supporting a maintained ncBAF complex interaction capacity of the alternative isoform. Moreover, the alternative BRD9 isoform showed a relatively higher interaction with TARBP1, and a relatively lower interaction with auxiliary factors, including SPEN, BRCA2, ASPM, and CHD9, that are known to interact with BRD9 [46]. *SPEN* has been described to be mutated in CLL and thought to suppress Notch signaling; a de-coupling of the BRD9 interaction with SPEN may potentially lead to a similar outcome [53]. In this context, it is relevant to mention that previously observed alternative splicing of *DVL2* has been linked to increased Notch signaling [3]. We also observed ASEs in *DVL2*, although they did not reach our cut-off. As for the BRCA2 interaction, it may be connected to the previously reported impaired DNA repair observed in *SF3B1*^*MUT*^ CLL [3]. Regarding differences in the integration of the two BRD9 splice isoforms into the ncBAF complex, co-immunoprecipitation experiments of BRD9 and BICRA, SMARCA4, and SMARCC1 revealed a higher affinity of the alternative BRD9 isoform to the ncBAF complex subunits.

In an integrative analysis of gene expression, chromatin accessibility, and DNA-protein interactions, we found that differentially expressed genes between *SF3B1*^*MUT*^ and *SF3B1*^*WT*^ are associated with an open chromatin structure in a focal region on chromosome 1, bound by BRD9 and the BAF complex subunits SMARCA4 and SMARCC2. Remarkably, the four adjacent genes located in this region, namely *NOL9*, *TAS1R1*, *ZBTB48*, and *KLHL21*, demonstrate higher expression levels in *SF3B1*^*MUT*^ CLL. Although their role in CLL pathogenesis is currently unknown, *ZBTB48* (also known as *TZAP*) is implicated in telomere maintenance whereby it binds to and shortens telomere length [54]. This is relevant, considering that particularly short telomeres and high telomerase activity have been reported in *SF3B1*^*MUT*^ and subset #2 patients [55, 56].

Increased BRD9 incorporation into the ncBAF complex has been reported in *SMARCB1*-mutated sarcoma cell lines [52] and demonstrated to induce synthetic lethality in synovial sarcoma and rhabdomyosarcoma cell lines with perturbed *SMARCB1* [44]. On these grounds, BRD9 targeting using PROTAC degraders is currently under investigation in a phase 1 clinical trial (NCT04965753) in patients with advanced synovial sarcoma or advanced *SMARCB1*-loss tumors.

In our BRD9 inhibition experiments, the *SF3B1*^*MUT*^ CLL and AML cell lines, CII and HNT34, respectively, were sensitive to I-BRD9, both regarding increased apoptosis and decreased proliferation rate. In addition, the PROTAC BRD9 Degrader-1 demonstrated anti-proliferative effects in both CII and HNT34. Furthermore, our DepMap analysis revealed the selective dependency of the CII cell line on *BRD9*, highlighting its critical role as a tumor suppressor. Intriguingly, the co-dependency of *BRD9* with *SMARCD1*, a BAF complex subunit, along with subunits of the Mediator and SAGA complexes, suggests an intricate interplay between chromatin remodeling complexes in CLL pathogenesis. Notably, *MED12*, a Mediator complex subunit, is recurrently mutated in CLL [57], and recent studies indicate crosstalk between the Mediator and BAF complexes [58].

Finally, in extended experiments using primary CLL cells, I-BRD9 treatment elicited comparable responses in *SF3B1*^*MUT*^ and *SF3B1*^*WT*^ cells in terms of IC_50_ values and pro-apoptotic effects. Thus, while treatment of cell lines revealed a distinct association with *SF3B1* mutations, primary CLL cells displayed sensitivity to I-BRD9 treatment independent of *SF3B1* mutation status. This finding implies a vulnerability to BRD9 inhibition in CLL that warrants further investigation.

In conclusion, we have identified distinct *SF3B1*^*MUT*^-related ASEs involving the ncBAF complex and characterized potential upstream and downstream mechanisms of action. These ASEs impact the ncBAF complex function from multiple vantage points with expected chromatin modulatory effects and subsequently altered gene expression. Our findings suggest functional consequences of *SF3B1* mutations and highlight ncBAF complex subunits as potential targets for therapeutic exploration in CLL.

## Supplementary information

Supplementary Information

Supplementary Tables

## Data Availability

The short-read and long-read RNA-sequencing data, including raw and aligned files, have been deposited at the European Genome-phenome Archive under the study accession EGAS00001006771. The direct long-read RNA-sequencing data of the CLL cell line HG3 have been deposited at Figshare with 10.17044/scilifelab.21694799.
