# Supplementary material for: The non-canonical BAF chromatin remodeling complex is a novel target of spliceosome dysregulation in SF3B1-mutated chronic lymphocytic leukemia
Source: Leukemia. 2024 Sep 11;38(11):2429–42. doi: 10.1038/s41375-024-02379-4 (PMC11518989; doi:10.1038/s41375-024-02379-4)
Supplement: Supplementary file 1 — Supplementary Information [file 41375_2024_2379_MOESM1_ESM.pdf]

Supplementary Information

The non-canonical BAF chromatin remodeling complex is a novel target of spliceosome dysregulation in *SF3B1*-mutated chronic lymphocytic leukemia

Daniel Hägerstrand*, Blaž Oder*, Diego Cortese, Ying Qu, Amrei Binzer-Panchal, Cecilia Österholm, Teresa Del Peso Santos, Leily Rabbani, Hassan Foroughi Asl, Aron Skaftason, Viktor Ljungström, August Lundholm, Maria Koutroumani, Zahra Haider, Cecilia Jylhä, John Mollstedt, Larry Mansouri, Karla Plevova, Andreas Agathangelidis, Lydia Scarfò, Marine Armand, Alice F Muggen, Neil E Kay, Tait Shanafelt, Davide Rossi, Lukas M Orre, Sarka Pospisilova, Konstantin Barylyuk, Frederic Davi, Mattias Vesterlund, Anton W Langerak, Janne Lehtiö, Paolo Ghia, Kostas Stamatopoulos, Lesley-Ann Sutton, Richard Rosenquist

*Contributed equally as first authors.

Alternative splicing, and differential gene expression analyses of short-read RNA-sequencing data

Acquired FASTQ files from RNA-sequencing (RNA-seq) were processed using the nf-core/rnaseq v1.0 pipeline with standard parameters[1]. The read quality was assessed using FastQC v0.11.7[2]. Reads were trimmed with Trim Galore v0.5.0 and aligned to the reference genome hg19 using STAR v2.6.1[3,4]. The 'MarkDuplicates' module of Picard v2.18.14 and dupRadar v1.8.0 were used to determine duplicate reads[5,6]. The quality of alignment was assessed with RSeQC v2.6.4, the 'CollectRnaSeqMetrics' module of Picard v2.18.14 (Supplementary Table 2A), and the 'flagstat' module of Samtools v1.16 (Supplementary Table 2B)[5,7,8]. Quality reports were generated using MultiQC v1.6[9]. Raw counts were retrieved using featureCounts[10]. Alternative splicing analysis was carried out using rMATS-turbo v4.1.2 using '--readLength' of 123, '--variable-read-length', and default parameters[11]. RNA-seq data was used to determine *SF3B1* mutation status. Variants were called following GATK best practices on RNA-seq variant calling[11]. Alternatively, variants were manually identified by loading BAM files into IGV v2.12.3[12] (Supplementary Table 1A).
Alternative splicing analysis was carried out using rMATS-turbo v4.1.2 using '--readLength' of 123, '--variable-read-length', and default parameters (Supplementary Table 3A-D)[13]. DESeq2 v1.38.3 was used to normalize the raw count data and determine gene expression values[14]. Sequencing batch effects and sex were controlled for during normalization. For cases lacking sex information, sex was inferred from the RNA-seq data. Immunoglobulin genes (acquired from the HUGO Gene Nomenclature Committee) were omitted from downstream analyses to prevent them from dominating subset-related comparisons[15,16].

Direct long-read RNA-sequencing of a CLL cell line

For direct long-read RNA-seq of the CLL cell line HG3 using Oxford Nanopore Technologies, total RNA was extracted using the RNAeasy kit (Qiagen, Hilden, Germany). Sequencing libraries were generated from ~5 µg of high-quality RNA (RIN > 9.7) as assessed by Agilent 2100 Bioanalyzer (Agilent Technologies, Santa Clara, CA, USA) and Direct RNA Sequencing kit (SQK-RNA002; Oxford Nanopore Technologies, Oxford, UK) was used according to the manufacturer's recommendations. Sequencing libraries were sequenced on a PromethION (Oxford Nanopore Technologies, Oxford, UK) using PromethION R9.4.1 flow cells (Oxford Nanopore Technologies, Oxford, UK).
Basecalling was performed using Guppy v6.1.2 and passed FASTQ files were analyzed using nf-core/nanoseq v1.1.0 pipeline[1]. The FASTQ files were aligned to the reference genome hg19 using Minimap2 v2.17[17]. IGV v2.12.3 was used for the visualization of splice variants and alternative splicing events (ASEs)[12].

Splice variant analysis using long-read RNA-sequencing

Long-read RNA-seq analysis using Oxford Nanopore Technologies was performed in 5 CLL cases, namely RS23 (*SF3B1*^{WT}), RS24 (*SF3B1*^{WT}), RS41 (*SF3B1*^{K700E}), RS55 (*SF3B1*^{G742D}), and RS62 (*SF3B1*^{K700E}). Total RNA was isolated from diagnostic patient samples as described above. Sequencing libraries were generated using the cDNA-PCR Sequencing Kit (SQK-

PCS109; Oxford Nanopore Technologies, Oxford, UK) according to the manufacturer's protocol. Sequencing was performed using MinION R9.4.1 flow cells (Oxford Nanopore Technologies, Oxford, UK) on a MinION sequencer (Oxford Nanopore Technologies, Oxford, UK) for 72 hours.

Basecalling was performed using Guppy v4.4.1 and the read quality was assessed by NanoComp v1.19.3 (Supplementary Table 2C) and FastQC v0.11.9[2,18]. The FASTQ files were aligned to the reference genome hg19 using Minimap2 v2.24 with '-ax splice' mode[17]. Subsequently, the following series of modules from Full-Length Alternative Isoform analysis of RNA (FLAIR) v1.6.4 were used to perform the alternative splicing analysis of our data and data downloaded from Tang et al.: 'bam2bed12' > 'flair correct' > 'flair collapse' > 'flair quantify' > 'flair diffSplice' (Supplementary Table 5)[19]. The outputs of the 'flair correct' module were merged into one file and used as input for the 'flair collapse' module while considering all the replicates and batches. This pipeline is available at https://github.com/clinicalgenetics/ont_cdna_pipeline (v0.0.1).

Cell culture

The CLL cell lines MEC1, PCL12, PGA1, HG3, and CII[20–24], the AML cell line HNT34[25], and the UVM cell line MEL202[26] were propagated in RPMI 1640 Medium (Gibco, Thermo Fischer Scientific, Waltham, MA, USA) supplemented with 10% heat-inactivated Fetal Bovine Serum (Gibco, Thermo Fischer Scientific, Waltham, MA, USA) and 0.2% MycoZap Plus-CL (Lonza, Basel, Switzerland). The HEK293T cell line was cultured in DMEM Medium with high glucose and GlutaMAX (Gibco, Thermo Fischer Scientific, Waltham, MA, USA) supplemented with 10% heat-inactivated Fetal Bovine Serum (Gibco, Thermo Fischer Scientific, Waltham, MA, USA), 1 mM sodium pyruvate (Gibco, Thermo Fischer Scientific, Waltham, MA, USA), 0.1 mM MEM Non-Essential Amino Acids Solution (Gibco, Thermo Fischer Scientific, Waltham, MA, USA), and 0.2% MycoZap Plus-CL (Lonza, Basel, Switzerland). All cell lines were maintained in a humidified incubator at 37 °C with 5% CO_2. Cell lines were authenticated using Eurofins's or Microsynth's cell line authentication service and routinely tested for mycoplasma contamination using Eurofins's Mycoplasmacheck service. The *SF3B1* mutation status in cell lines was confirmed by Sanger sequencing.

Inducible SF3B1^{K700E} and SF3B1^{WT} CLL cell lines

The sequences of the wildtype and mutated *SF3B1* variants have undergone codon optimization and correspond to the sequences in the plasmids pCDNA3.1-FLAG-SF3B1-WT (Addgene plasmid #82576; http://n2t.net/addgene:82576; RRID: Addgene_82576) and pCDNA3.1-FLAG-hSF3B1-K700E (Addgene plasmid #82577; http://n2t.net/addgene:82577; RRID: Addgene_82577), a gift from Manoj Pillai[27]. *SF3B1*^{K700E} and *SF3B1*^{WT} variants were cloned into the lentiviral transfer plasmid pCW57.1, a gift from David Root (Addgene plasmid #41393; http://n2t.net/addgene:41393; RRID: Addgene_41393), and lentiviral vectors were generated as previously described[28]. The CLL cell lines MEC1 and PCL12 were transduced and exogenous *SF3B1* overexpression was induced with 2 µg/ml of doxycycline (Merck, Darmstadt, Germany) for 7 days, including renewal of medium and doxycycline after 4 days.

PCR analysis of BRD9 splice variants

Total RNA was extracted using the RNeasy Mini Kit (Qiagen, Hilden, Germany), and cDNA was generated with the Maxima H Minus First Strand cDNA Synthesis Kit, with dsDNase (Thermo Fisher Scientific, Waltham, MA, USA) according to the instructions by the manufacturer. Transcript levels were determined by qPCR using the PowerUp SYBR Green Master Mix (Applied Biosystems, Thermo Fisher Scientific, Waltham, MA, USA) on a CFX96 Touch Real-Time PCR Detection System (Bio-Rad Laboratories, Hercules, CA, USA). The following primer combinations were used; endogenous *SF3B1*, AAGCAAGAAGTCCTGGCAAG and AAAGCAGCCAAACCCTTTCC, exogenous *SF3B1*, ACACCATACGGGATCGAGAG and GCACTGCTTGACCACTTTCA, *BRD9* transcripts in general, TTGGGGACACCCTAGGAGAC and CAGAGAGCTGAGCATGGAGA, regular *BRD9* transcript, CAGCAGCTCTGTTCTGGAGT and TCACCTTCCCCAGAGAGCTG, alternative *BRD9* transcript, CAGCAGCTCTGTTCTGGAGT and TCACCTTCCCCAGAGAGCTG. *GAPDH* levels were used for the normalization of cDNA input with the TCATTTCCTGGTATGACAACGA and GTCTTACTCCTTGGAGGCC primer pair. The presence of the regular and alternative *BRD9* transcripts was also assessed by PCR using the REDTaq ReadyMix PCR Reaction Mix (Sigma-Aldrich, Merck, Darmstadt, Germany) with a non-transcript-specific primer pair TTGGGGACACCCTAGGAGAC and GTCCAGCTCCTTCTTCACCT. Reactions were run on a SimpliAmp Thermal Cycler (Applied Biosystems, Thermo Fisher Scientific, Waltham, MA, USA) and the PCR products were separated by agarose gel electrophoresis. For a complete gel image see Supplementary Figure 13A.

Cloning of BRD9 splice variants and generation of stable cell lines

Total RNA, from the MEC1 cell line and an $SF3B1^{MUT}$ CLL case, which expressed relatively high levels of the alternative *BRD9* transcripts as assessed from the RNA-seq data, was extracted using the RNeasy Mini Kit (Qiagen, Hilden, Germany), and cDNA was generated using the Maxima H Minus First Strand cDNA Synthesis Kit, with dsDNase (Thermo Fisher Scientific, Waltham, MA, USA) according to the instructions by the manufacturer. PCR amplification of *BRD9* transcripts was performed with Platinum SuperFi II Green PCR Master Mix (Invitrogen, Thermo Fisher Scientific, Waltham, MA, USA) using transcript-specific primers to generate fragments for downstream Gateway cloning. The primers contained attB sequence overhangs to allow for BP Clonase ligation as previously described[29]. Two *BRD9* splice variants were selectively amplified, the regular splice variant from the MEC1 cells and the alternative splice variant with the alternative exon from the $SF3B1^{MUT}$ CLL case. For both splice variants the same forward primer was used, GGGGACAACTTTGTACAAAAAAGTTGGCATGGGCAAGAAGCACAAGAAG (*BRD9*-specific sequence underlined). For the regular *BRD*9 splice variant reverse primer GGGGACAACTTTGTACAAGAAAGTTGGGTAGGTCTTGGCAGAGGCCG (*BRD9*-specific sequence underlined) and for the alternative *BRD9* splice variant GGGGACAACTTTGTACAAGAAAGTTGGGCAGGCACAGCTGACCAGC (*BRD9*-specific sequence underlined) were used. PCR products were separated and controlled by agarose gel electrophoresis and purified using the QIAquick Gel Extraction Kit (Qiagen,

Hilden, Germany). Subsequently, the PCR products were ligated into the entry plasmid pDONR223 to generate entry clones that were subjected to screening by colony PCR with M13- and exon-specific primers[30]. Potential clones were analyzed by Sanger sequencing (Appendix 1). For each splice variant, one desired clone was selected for downstream Gateway LR Clonase ligation into the lentiviral transfer plasmid pLenti6.2-3xFLAG-V5-ccdB (Addgene plasmid #87072; http://n2t.net/addgene:87072; RRID: Addgene_87072), a gift from Susan Lindquist and Mikko Taipale[30], to create an expression clone. Lentiviral vectors were generated as previously described and the HEK293T cell line was transduced for stable overexpression of FLAG-V5-tagged *BRD9* splice variants[28]. Control cell lines were transduced with the same viral backbone as empty or overexpressing FLAG-V5-tagged yellow fluorescent protein (YFP).

Cell lysis, SDS-PAGE, Western blotting, and antibodies

Cell lysates were generated by lysis of pelleted Dulbecco's PBS (DPBS)-washed cells in ice-cold lysis buffer (25 mM Tris-HCl, 150 mM NaCl, 2 mM EDTA, 1% NP-40, and 5% glycerol; pH 7.4) supplemented with proteinase and phosphatase inhibitors, cOmplete Protease Inhibitor Cocktail (Roche, Basel, Switzerland) and PhosSTOP (Roche, Basel, Switzerland), respectively. Proteins were quantified by BCA Protein Assay Kit (Pierce, Thermo Fisher Scientific, Waltham, MA, USA), separated by SDS-PAGE using Mini-PROTEAN TGX precast gels (Bio-Rad Laboratories, Hercules, CA, USA), and transferred to PVDF membranes using a Trans-Blot Turbo Transfer System (Bio-Rad Laboratories, Hercules, CA, USA). Western blot detection was performed with Clarity Max Western ECL Substrate (Bio-Rad Laboratories, Hercules, CA, USA) or SuperSignal West Femto Maximum Sensitivity Substrate (Thermo Fisher Scientific, Waltham, MA, USA) on a ChemiDoc Imaging System (Bio-Rad Laboratories, Hercules, CA, USA). Rabbit anti-BRD9 antibody raised against amino acids 323-411 (HPA021465; Sigma-Aldrich, Merck, Darmstadt, Germany), rabbit anti-BRD9 antibody raised against amino acids 547-597 (A303-781A; Bethyl Laboratories, Montgomery, TX, USA), mouse anti-GLTSCR1 (BICRA) antibody (sc-515086; Santa Cruz Biotechnology, Dallas, TX, USA), mouse anti-BRG1 (SMARCA4) antibody (sc-17796; Santa Cruz Biotechnology, Dallas, TX, USA), mouse anti-BAF155 (SMARCC1) antibody (sc-48350; Santa Cruz Biotechnology, Dallas, TX, USA), and mouse anti-V5 tag antibody (R960-25; Invitrogen, Thermo Fisher Scientific, Waltham, MA, USA) were utilized in combination with a corresponding secondary antibody, donkey anti-rabbit HRP-conjugated antibody (NA934; Cytiva, Marlborough, MA, USA) or sheep anti-mouse HRP-conjugated antibody (NA931; Cytiva, Marlborough, MA, USA). Mouse anti-beta actin HRP-conjugated antibody (sc-47778; Santa Cruz Biotechnology, Dallas, TX, USA) was used for the assessment of loaded protein levels.

Co-immunoprecipitation

Nuclear extracts were prepared by NE-PER Nuclear and Cytoplasmic Extraction Reagents (Thermo Fisher Scientific, Waltham, MA, USA) and incubated with an appropriate amount of antibody as recommended by the manufacturers. In general, 1-2 µg of an antibody was used per 100-200 µg of nuclear extract per immunoprecipitation. Immune complexes were captured

with Dynabeads Protein G (Invitrogen, Thermo Fisher Scientific, Waltham, MA, USA) and eluted with SDS-PAGE sample loading buffer. Raw values for the calculations in Figure 4G and complete blot images from these experiments can be found in Supplementary Figure 13A, B.

Mass spectrometry analysis of co-immunoprecipitates

The immunoprecipitation beads were reconstituted in 100 µl of HEPES buffer solution (pH 7.6) containing 1 mM DTT. The bead-bound proteins were reduced with 1 mM DTT for 30 minutes and thereafter alkylated in 5 mM chloroacetamide in the dark for 20 minutes. The remaining chloroacetamide was quenched by the addition of DTT to a final concentration of 5 mM. Digestion was carried out by the addition of 0.4 µg MS-grade Trypsin Protease (Pierce, Thermo Fisher Scientific, Waltham, MA, USA) and overnight incubation at 37 °C. The supernatant was collected and reduced to dryness in a SpeedVac vacuum centrifuge (Thermo Fisher Scientific, Waltham, MA, USA). The peptides were reconstituted in 40 µl of 50 mM HEPES buffer solution (pH 7.6) and cleaned by a modified sp3 protocol[31]. Briefly, 10 µl of Sera-Mag SP3 bead mix (10 µg/µl) was added to the sample and 100% acetonitrile was added to achieve a final concentration of > 95%. Samples were incubated on a rotational mixer for 30 minutes, the supernatants were aspirated, and the beads were washed twice in 1000 µl of acetonitrile. The beads were reconstituted in 100 µl of the sample loading solution (0.1% formic acid and 3% aqueous acetonitrile). The supernatants were recovered, transferred to LC-MS sample vials, reduced to dryness on a vacuum centrifuge, and reconstituted in 15 µl of the sample loading solution.

Online LC-MS/MS was performed using a Dionex Ultimate 3000 RSLCnano System (Thermo Fisher Scientific, Waltham, MA, USA) coupled to a Q Exactive mass spectrometer (Thermo Fisher Scientific, Waltham, MA, USA). Five µl was injected from each sample. Samples were trapped on a C18 guard desalting column (Acclaim PepMap 100, NanoViper, C18, 5 µm, 100 Å, 75 µm x 2 cm; Thermo Fisher Scientific, Waltham, MA, USA), and separated on an analytical C18 column (EASY-Spray PepMap RSLC, C18, 2 µm, 100 Å, 75 µm x 15 cm; Thermo Fisher Scientific, Waltham, MA, USA). The nano capillary solvent A was 95% water, 5% DMSO, and 0.1% formic acid; and solvent B was 5% water, 5% DMSO, 95% acetonitrile, and 0.1% formic acid. At a constant flow of 0.25 µl/min, the curved gradient went from 6% B up to 43% B in 180 minutes, followed by a steep increase to 100% B in 5 minutes.

FTMS master scans with a resolution of 60,000 and a mass range of 300-1500 m/z were followed by data-dependent MS/MS with a resolution of 30,000 on the top 5 ions using higher energy collision dissociation at 30% normalized collision energy. Precursors were isolated with a 2 m/z window. Automatic gain control targets were 1,000,000 for MS1 and 100,000 for MS2. Maximum injection time was 100 milliseconds for MS1 and MS2. The entire duty cycle lasted ~ 2.5 seconds. Dynamic exclusion was used with a 60-second duration. Precursors with unassigned charge state or charge state 1 were excluded. The underfill ratio of 1% was used.

The processing of raw LC-MS data for peptide and protein identification and quantification was performed with Proteome Discoverer v2.1 (Thermo Fisher Scientific, Waltham, MA, USA). Raw mass spectra were filtered, converted to peak lists by Proteome Discoverer, and submitted to a database search using the Sequest HT search engine against the canonical and

isoform protein sequences of *Homo sapiens* (42,367 entries retrieved from UniProt on 2020-01-16) and common contaminant proteins (370 proteins retrieved from https://github.com/HaoGroup-ProtContLib)[31]. The sequences of bait proteins were included in the search database for the corresponding samples. The precursor and fragment mass tolerances were set to 10 ppm and 0.02 Da, respectively. The enzyme was set to trypsin with up to two missed cleavages allowed. Carbamidomethylation of cysteine was set as a static modification. Oxidation of methionine, the acetylation of protein N-terminus, the loss of protein N-terminal methionine, and the combination of the N-terminal methionine loss and acetylation were set as dynamic modifications. The false discovery rate (FDR) of peptide-to-spectrum matches (PSMs) was validated by Percolator v2.05 and only high-confidence peptides (FDR < 0.01) of a minimum length of 6 amino acid residues were used for protein identification[32]. The processing results of individual LC-MS files were summarized in a consensus workflow to group, score, filter, and validate peptides and proteins, and compute protein FDR. Strict parsimony was applied for protein grouping. Chromatographic peak areas of identified peptides were determined from the respective precursor ion extracted ion chromatograms. Unique peptides were considered for protein quantification using an average abundance of the top 3 most abundant peptides. The protein level report was exported from Proteome Discoverer and used as input for subsequent data analysis (Supplementary Table 8A-C).

Analysis of publicly available data

RNA-seq data, approved by the International Cancer Genome Consortium's Data Access Compliance Office (ICGC DACO), application DACO-6103, from the Chronic Lymphocytic Leukemia - ISC/MICINN, ES (CLLE-ES) provided by the Spanish consortium, was downloaded for 74 CLL cases via the ICGC portal (Supplementary Table 1B)[33,34]. The ICGC CLLE-ES RNA-seq data was processed similarly as described above for our CLL cases to determine the occurrence of splice variants (Supplementary Table 6A, B).
For The Cancer Genome Atlas (TCGA) CLL gene expression data from the Broad study from 2015, a gene expression matrix expressed as TPM values (file 'data_mrna_seq_tpm.txt') and mutation data (file 'data_mutations.txt') were downloaded from the cBioPortal (http://www.cbioportal.org) (Supplementary Table 1C)[35–38]. Substantial batch effects were observed between cases with IDs beginning with 'ICGC_*' and 'DFCI-*'. To correct these batch effects, the log_2-transformed TPM counts were processed using the 'removeBatchEffect' module of limma v3.54.2[39]. Subsequently, differential gene expression analysis between *SF3B1*^{MUT} and *SF3B1*^{WT} cases was conducted using the limma-trend approach[39].
For the Cancer Cell Line Encyclopedia (CCLE) cell lines, exon usage data, *BRD9* dependency data, and *SF3B1* mutation status were retrieved via the Cancer Dependency Map (DepMap) portal (https://depmap.org, files 'CCLE_RNAseq_ExonUsageRatio_20180929.gct' from CCLE 2019 edition (1,019 cancer cell lines), 'CRISPR_gene_effect.csv' (1,086 cancer cell lines), and 'CCLE_mutations.csv' from the 22Q2 edition)[40,41]. Exon usage for the corresponding ASEs in transcripts encoding ncBAF complex-related proteins was extracted and used for groupwise statistical comparisons. Based on sequencing data for *SF3B1*, PANC0504 (p.699_700QK>HE), HNT34 (p.K700E), MUTZ3 (p.K666N), and ESS1 (p.K666N) were considered as *SF3B1*^{MUT} cell lines. These cell lines originate from the

following cancer types, PANC0504 pancreatic cancer, HNT34 and MUTZ3 acute myeloid leukemia, and ESS1 endometrial stromal sarcoma.

The long-read RNA-seq data from Tang et al. deposited at dbGaP (study accession phs001959.v1.p1) was downloaded by the SRA Toolkit[19,42]. This included 4 *SF3B1*^{WT} (SRR1142437, SRR1142440, SRR1142441, and SRR1142442) and 4 *SF3B1*^{MUT} CLL cases (SRR1142443, SRR1142444, SRR1142445, and SRR1142446). SRR1142437 and SRR1142443 have been sequenced on the MinION platform, and the remaining ones on the PromethION platform.

The short-read RNA-seq data for MEL202 (SRR12354765) and HNT34 (SRR8616208) were downloaded by the SRA Toolkit[43,44]. The read quality was assessed using FastQC v0.11.9 and the FASTQ files were mapped to the reference genome hg19 by STAR v2.7.8a[2,4].

The proteomic expression data from CLL produced by Herbst et al. was retrieved from ProteomeXchange (accession number PXD028936)[45].

For analysis of histone marks and BRD9, SMARCA4, SMARCC2, and CTCF chromatin binding, publicly available ChIP-seq data was downloaded from the ENCODE database (https://www.encodeproject.org) for the chronic myeloid leukemia cell line K562 regarding precipitations targeting H3K27ac (ENCFF840LLW), H3K4me3 (ENCFF285EUA), H3K9ac (ENCFF957FQF), H3K79me2 (ENCFF677DJM), H3K36me3 (ENCFF464YSK), H3K27me3 (ENCFF658JMW), H3K9me3 (ENCFF330EOT), BRD9 (ENCFF787SKU), SMARCA4 (ENCFF220ZHN), SMARCC2 (ENCFF699USV), and CTCF (ENCFF468HJA)[46,47].

Assessment of BRD9 drug sensitivity in CLL cell lines and primary CLL cells

The BRD9 inhibitor I-BRD9 (SML1534; Sigma-Aldrich, Merck, Darmstadt, Germany) and PROTAC BRD9 Degrader-1 (HY-103632; MedChemExpress, NJ, USA) were dissolved in DMSO to obtain 10 mM stock solutions[48,49]. The BRD9 degrader dBRD9 Hydrochloride (SML2911, Sigma-Aldrich, Merck, Darmstadt, Germany) was dissolved in water to obtain a 2.55 mM stock solution[48]. For dose-response analysis, cell lines and primary CLL cells were cultured in 96-well plates for 3 and 2 days, respectively, in the presence of the inhibitors/degraders at concentrations ranging from 0.001 to 50 µM. Relative amounts of metabolically active cells were determined with CellTiter-Glo 2.0 (Promega, Madison, WI, USA).

Assessment of proliferation and apoptosis was performed using the APC BrdU Kit (BD Pharmingen, BD Biosciences, Franklin Lakes, NJ, USA) and FITC Annexin V Apoptosis Detection Kit I (BD Pharmingen, BD Biosciences, Franklin Lakes, NJ, USA), respectively, both according to the manufacturer´s instructions. For proliferation assessment, positive controls were treated with 5 µM Camptothecin, while all other conditions, except the negative controls treated with vehicle (DMSO), were exposed to 10 µM BrdU 5 hours prior to cell harvest. DPBS-washed cells were collected for fixation, cryopreservation, and subsequent assessment of BrdU incorporation. Non-BrdU-exposed cells were used to set the baseline for proliferation. BrdU+ cells were regarded as proliferating cells. For apoptosis assessment, the cells were resuspended in 100 µl of Annexin V binding buffer and thereafter 5 µl of Annexin V and 5 µl of Propidium Iodide (PI) were added followed by incubation in the dark for 15 minutes prior to flow cytometry. Camptothecin-treated samples stained with either Annexin V

or PI were used for compensation purposes and gating. Annexin V+/PI- and Annexin V+/PI+ cells were regarded as early and late apoptotic, respectively. All samples were acquired on a BD Accuri C6 Plus Flow Cytometer (BD Biosciences, Franklin Lakes, NJ, USA).

Statistics

The normality of the data distribution and variance were examined using the Shapiro-Wilk test and Bartlett's test, respectively. Based on these assessments, appropriate statistical tests, such as Student's *t*-test or Wilcoxon rank-sum test, were performed to determine the significance of differences between groups. Benjamini-Hochberg multiple testing correction was applied for multiple comparisons. One-way ANOVA with Dunnett's post-hoc test was used to compare the means of three or more independent groups. Binomial tests were used to determine the significance of outcomes. Pearson correlation coefficient and logistic regression were used to determine the correlation between parameters. Clustering was conducted using the Ward method and the Euclidean metric. Dose-response curve fitting was performed using four-parameter logistic regression. Western blot protein quantification was carried out using Fiji v2.9.0[50]. Data processing and statistical analyses were conducted using Prism 9 (GraphPad Software, San Diego, CA, USA), Microsoft Excel (Microsoft, Redmond, WA, USA), RStudio v2022.7.0.548 with R v4.2.2 and packages stats v4.4.0 and drc v3.0.1[51–53], and Jupyter Notebook v7.0.6 with packages NumPy v1.26.3, Pandas v2.1.4, and SciPy v1.11.4[54–57].

Figures

Scattered box plots, bar plots, scatter plots, volcano plots, network plots, dose-response curves, clustermaps, and heatmaps were created using RStudio v2022.7.0.548 with R v4.2.2 and packages ggplot2 v3.5.0 and drc v3.0.1[51–53,58], and Jupyter Notebook v7.0.6 with packages Seaborn v0.13.1, Matplotlib v3.8.2, and NetworkX v3.2.1[57,59–61]. Sashimi plots were generated using the sashimi visualization function in IGV v2.12.3[12]. Flow cytometry data was visualized using FlowJO v10.9.0 (BD Biosciences, Franklin Lakes, NJ, USA). Final preparation of images was performed in Adobe Illustrator 2023 (Adobe, Mountain View, CA, USA) and Adobe Photoshop 2023 (Adobe, Mountain View, CA, USA). The gene names as they appear in the Figures may not match those in the Supplementary Tables as they reflect the most recent official gene names according to the HUGO Gene Nomenclature Committee.

References

1. Ewels PA, Peltzer A, Fillinger S, Patel H, Alneberg J, Wilm A *et al.* The nf-core framework for community-curated bioinformatics pipelines. *Nat Biotechnol* 2020; **38**: 276–278.
2. Andrews S. FastQC: A Quality Control Tool for High Throughput Sequence Data. 2010.http://www.bioinformatics.babraham.ac.uk/projects/fastqc/.
3. Krueger F. Trim Galore. 2015.https://www.bioinformatics.babraham.ac.uk/projects/trim_galore/.
4. Dobin A, Davis CA, Schlesinger F, Drenkow J, Zaleski C, Jha S *et al.* STAR: ultrafast universal RNA-seq aligner. *Bioinformatics* 2013; **29**: 15–21.
5. Broad Institute. Picard Tools. 2019.http://broadinstitute.github.io/picard/.
6. Sayols S, Scherzinger D, Klein H. dupRadar: a Bioconductor package for the assessment of PCR artifacts in RNA-Seq data. *BMC Bioinformatics* 2016; **17**: 428.
7. Wang L, Wang S, Li W. RSeQC: quality control of RNA-seq experiments. *Bioinformatics* 2012; **28**: 2184–2185.
8. Li H, Handsaker B, Wysoker A, Fennell T, Ruan J, Homer N *et al.* The Sequence Alignment/Map format and SAMtools. *Bioinformatics* 2009; **25**: 2078–2079.
9. Ewels P, Magnusson M, Lundin S, Käller M. MultiQC: summarize analysis results for multiple tools and samples in a single report. *Bioinformatics* 2016; **32**: 3047–3048.
10. Liao Y, Smyth GK, Shi W. featureCounts: an efficient general purpose program for assigning sequence reads to genomic features. *Bioinformatics* 2014; **30**: 923–930.
11. Van der Auwera GA, Carneiro MO, Hartl C, Poplin R, del Angel G, Levy-Moonshine A *et al.* From FastQ Data to High-Confidence Variant Calls: The Genome Analysis Toolkit Best Practices Pipeline. *Curr Protoc Bioinformatics* 2013; **43**: 11.10.1-11.10.33.
12. Robinson JT, Thorvaldsdóttir H, Winckler W, Guttman M, Lander ES, Getz G *et al.* Integrative genomics viewer. *Nat Biotechnol* 2011; **29**: 24–26.
13. Shen S, Park JW, Lu Z, Lin L, Henry MD, Wu YN *et al.* rMATS: Robust and flexible detection of differential alternative splicing from replicate RNA-Seq data. *Proceedings of the National Academy of Sciences* 2014; **111**: E5593–E5601.
14. Love MI, Huber W, Anders S. Moderated estimation of fold change and dispersion for RNA-seq data with DESeq2. *Genome Biol* 2014; **15**: 550.
15. Seal RL, Braschi B, Gray K, Jones TEM, Tweedie S, Haim-Vilmovsky L *et al.* Genenames.org: the HGNC resources in 2023. *Nucleic Acids Res* 2023; **51**: D1003–D1009.
16. Lefranc M-P. Immunoglobulin and T Cell Receptor Genes: IMGT® and the Birth and Rise of Immunoinformatics. *Front Immunol* 2014; **5**: 22–22.
17. Li H. Minimap2: pairwise alignment for nucleotide sequences. *Bioinformatics* 2018; **34**: 3094–3100.
18. De Coster W, D'Hert S, Schultz DT, Cruts M, Van Broeckhoven C. NanoPack: visualizing and processing long-read sequencing data. *Bioinformatics* 2018; **34**: 2666–2669.

19 Tang AD, Soulette CM, van Baren MJ, Hart K, Hrabeta-Robinson E, Wu CJ *et al.* Full-length transcript characterization of SF3B1 mutation in chronic lymphocytic leukemia reveals downregulation of retained introns. *Nat Commun* 2020; **11**: 1438.

20 Stacchini A, Aragno M, Vallario A, Alfarano A, Circosta P, Gottardi D *et al.* MEC1 and MEC2: two new cell lines derived from B-chronic lymphocytic leukaemia in prolymphocytoid transformation. *Leuk Res* 1999; **23**: 127–136.

21 Karande A, Fialkow PJ, Nilsson K, Povey S, Klein G, Najfeld V *et al.* Establishment of a lymphoid cell line from leukemic cells of a patient with chronic lymphocytic leukemia. *Int J Cancer* 1980; **26**: 551–556.

22 Rosén A, Bergh AC, Gogolák P, Evaldsson C, Myhrinder AL, Hellqvist E *et al.* Lymphoblastoid cell line with B1 cell characteristics established from a chronic lymphocytic leukemia clone by in vitro EBV infection. *Oncoimmunology* 2012; **1**: 18–27.

23 Lewin N, Åman P, Mellstedi H, Zech L, Klein G. Direct outgrowth of in vivo epstein-barr virus (EBV)-infected chronic lymphocytic leukemia (CLL) cells into permanent lines. *Int J Cancer* 1988; **41**: 892–895.

24 Agathangelidis A, Scarfò L, Barbaglio F, Apollonio B, Bertilaccio MTS, Ranghetti P *et al.* Establishment and Characterization of PCL12, a Novel CD5+ Chronic Lymphocytic Leukaemia Cell Line. *PLoS One* 2015; **10**: e0130195.

25 Hamaguchi H, Suzukawa K, Nagata K, Yamamoto K, Yagasaki F, Morishita K. Establishment of a novel human myeloid leukaemia cell line (HNT-34) with t(3;3)(q21;q26), t(9;22)(q34;q11) and the expression of EVI1 gene, P210 and P190 BCR/ABL chimaeric transcripts from a patient with AML after MDS with 3q21q26 syndrome. *Br J Haematol* 1997; **98**: 399–407.

26 Ksander BR, Rubsamen PE, Olsen KR, Cousins SW, Streilein JW. Studies of tumor-infiltrating lymphocytes from a human choroidal melanoma. *Invest Ophthalmol Vis Sci* 1991; **32**: 3198–3208.

27 Kesarwani AK, Ramirez O, Gupta AK, Yang X, Murthy T, Minella AC *et al.* Cancer-associated SF3B1 mutants recognize otherwise inaccessible cryptic 3′ splice sites within RNA secondary structures. *Oncogene 2017 36:8* 2016; **36**: 1123–1133.

28 Moffat J, Grueneberg DA, Yang X, Kim SY, Kloepfer AM, Hinkle G *et al.* A Lentiviral RNAi Library for Human and Mouse Genes Applied to an Arrayed Viral High-Content Screen. *Cell* 2006; **124**: 1283–1298.

29 Rual JF, Hirozane-Kishikawa T, Hao T, Bertin N, Li S, Dricot A *et al.* Human ORFeome Version 1.1: A Platform for Reverse Proteomics. *Genome Res* 2004; **14**: 2128–2135.

30 Taipale M, Tucker G, Peng J, Krykbaeva I, Lin ZY, Larsen B *et al.* A quantitative chaperone interaction network reveals the architecture of cellular protein homeostasis pathways. *Cell* 2014; **158**: 434.

31 Frankenfield AM, Ni J, Ahmed M, Hao L. Protein Contaminants Matter: Building Universal Protein Contaminant Libraries for DDA and DIA Proteomics. *J Proteome Res* 2022; **21**: 2104–2113.

32 The M, MacCoss MJ, Noble WS, Käll L. Fast and Accurate Protein False Discovery Rates on Large-Scale Proteomics Data Sets with Percolator 3.0. *J Am Soc Mass Spectrom* 2016; **27**: 1719–1727.
33 Zhang J, Bajari R, Andric D, Gerthoffert F, Lepsa A, Nahal-Bose H *et al.* The International Cancer Genome Consortium Data Portal. *Nat Biotechnol* 2019; **37**: 367–369.
34 Ramsay AJ, Martínez-Trillos A, Jares P, Rodríguez D, Kwarciak A, Quesada V. Next-generation sequencing reveals the secrets of the chronic lymphocytic leukemia genome. *Clinical and Translational Oncology* 2013; **15**: 3–8.
35 Landau DA, Tausch E, Taylor-Weiner AN, Stewart C, Reiter JG, Bahlo J *et al.* Mutations driving CLL and their evolution in progression and relapse. *Nature* 2015; **526**: 525–530.
36 Aaltonen LA, Abascal F, Abeshouse A, Aburatani H, Adams DJ, Agrawal N *et al.* Pan-cancer analysis of whole genomes. *Nature* 2020; **578**: 82–93.
37 Gao J, Aksoy BA, Dogrusoz U, Dresdner G, Gross B, Sumer SO *et al.* Integrative analysis of complex cancer genomics and clinical profiles using the cBioPortal. *Sci Signal* 2013; **6**: 1–1.
38 Cerami E, Gao J, Dogrusoz U, Gross BE, Sumer SO, Aksoy BA *et al.* The cBio Cancer Genomics Portal: An Open Platform for Exploring Multidimensional Cancer Genomics Data. *Cancer Discov* 2012; **2**: 401–404.
39 Ritchie ME, Phipson B, Wu D, Hu Y, Law CW, Shi W *et al.* limma powers differential expression analyses for RNA-sequencing and microarray studies. *Nucleic Acids Res* 2015; **43**: e47–e47.
40 Barretina J, Caponigro G, Stransky N, Venkatesan K, Margolin AA, Kim S *et al.* The Cancer Cell Line Encyclopedia enables predictive modelling of anticancer drug sensitivity. *Nature* 2012; **483**: 603–607.
41 Tsherniak A, Vazquez F, Montgomery PG, Weir BA, Kryukov G, Cowley GS *et al.* Defining a Cancer Dependency Map. *Cell* 2017; **170**: 564-576.e16.
42 Mailman MD, Feolo M, Jin Y, Kimura M, Tryka K, Bagoutdinov R *et al.* The NCBI dbGaP database of genotypes and phenotypes. *Nat Genet* 2007; **39**: 1181–1186.
43 Patel RP, Thomas JR, Curt KM, Fitzsimmons CM, Batista PJ, Bates SE *et al.* Dual Inhibition of Histone Deacetylases and the Mechanistic Target of Rapamycin Promotes Apoptosis in Cell Line Models of Uveal Melanoma. *Invest Ophthalmol Vis Sci* 2021; **62**: 16–16.
44 Ghandi M, Huang FW, Jané-Valbuena J, Kryukov G V., Lo CC, McDonald ER *et al.* Next-generation characterization of the Cancer Cell Line Encyclopedia. *Nature 2019 569:7757* 2019; **569**: 503–508.
45 Herbst SA, Vesterlund M, Helmboldt AJ, Jafari R, Siavelis I, Stahl M *et al.* Proteogenomics refines the molecular classification of chronic lymphocytic leukemia. *Nat Commun* 2022; **13**: 6226.
46 Luo Y, Hitz BC, Gabdank I, Hilton JA, Kagda MS, Lam B *et al.* New developments on the Encyclopedia of DNA Elements (ENCODE) data portal. *Nucleic Acids Res* 2020; **48**: D882–D889.

47 ENCODE Project Consortium. An integrated encyclopedia of DNA elements in the human genome. *Nature* 2012; **489**: 57–74.
48 Remillard D, Buckley DL, Paulk J, Brien GL, Sonnett M, Seo H *et al.* Degradation of the BAF Complex Factor BRD9 by Heterobifunctional Ligands. *Angewandte Chemie International Edition* 2017; **56**: 5738–5743.
49 Theodoulou NH, Bamborough P, Bannister AJ, Becher I, Bit RA, Che KH *et al.* Discovery of I-BRD9, a Selective Cell Active Chemical Probe for Bromodomain Containing Protein 9 Inhibition. *J Med Chem* 2016; **59**: 1425–1439.
50 Schindelin J, Arganda-Carreras I, Frise E, Kaynig V, Longair M, Pietzsch T *et al.* Fiji: an open-source platform for biological-image analysis. *Nature Methods 2012 9:7* 2012; **9**: 676–682.
51 R Core Team. R: A Language and Environment for Statistical Computing. 2022.https://www.R-project.org/.
52 Ritz C, Baty F, Streibig JC, Gerhard D. Dose-Response Analysis Using R. *PLoS One* 2015; **10**: e0146021.
53 RStudio Team. RStudio: Integrated Development Environment for R. 2022.http://www.rstudio.com/.
54 Harris CR, Millman KJ, van der Walt SJ, Gommers R, Virtanen P, Cournapeau D *et al.* Array programming with NumPy. *Nature 2020 585:7825* 2020; **585**: 357–362.
55 McKinney W. Data Structures for Statistical Computing in Python. In: *Proceedings of the 9th Python in Science Conference*. SciPy, 2010, pp 56–61.
56 Virtanen P, Gommers R, Oliphant TE, Haberland M, Reddy T, Cournapeau D *et al.* SciPy 1.0: fundamental algorithms for scientific computing in Python. *Nat Methods* 2020; **17**: 261–272.
57 Kluyver T, Ragan-Kelley B, Pérez F, Granger B, Bussonnier M, Frederic J *et al.* Jupyter Notebooks – a publishing format for reproducible computational workflows. In: *Positioning and Power in Academic Publishing: Players, Agents and Agendas*. IOS Press, 2016, pp 87–90.
58 Wickham H. *ggplot2: Elegant Graphics for Data Analysis*. Springer-Verlag New York, 2016.
59 Waskom M. seaborn: statistical data visualization. *J Open Source Softw* 2021; **6**: 3021.
60 Hunter JD. Matplotlib: A 2D graphics environment. *Comput Sci Eng* 2007; **9**: 90–95.
61 Hagberg A, Swart P, S Chult D. Exploring network structure, dynamics, and function using NetworkX. In: *Proceedings of the 7th Python in Science Conference*. SciPy, 2008, pp 11–16.
62 Stark C, Breitkreutz B-J, Reguly T, Boucher L, Breitkreutz A, Tyers M. BioGRID: a general repository for interaction datasets. *Nucleic Acids Res* 2006; **34**: D535–D539.

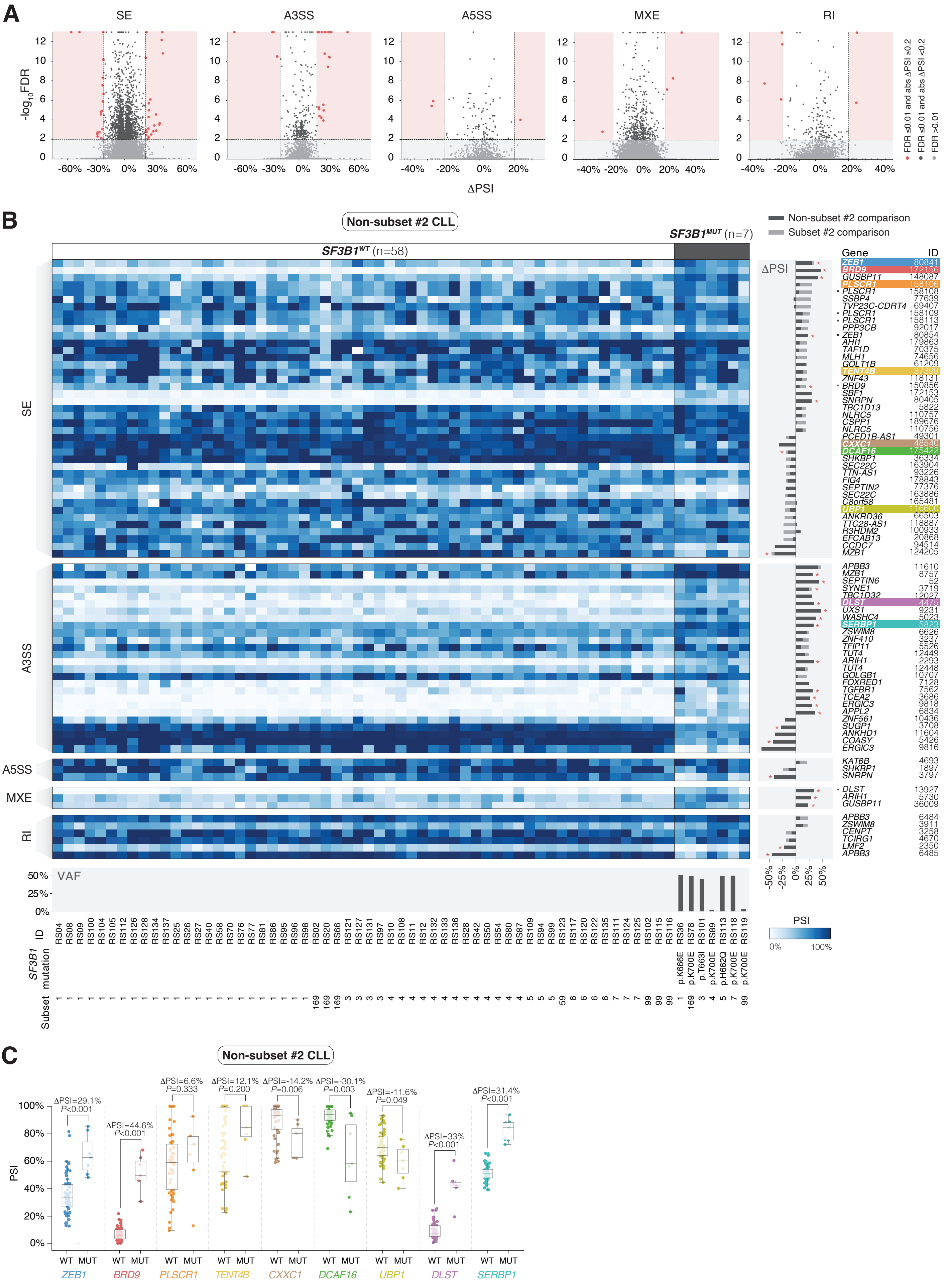

A
SE
A3SS
A5SS
MXE
RI
-log10FDR
ΔPSI
FDR ≤0.01 and abs ΔPSI ≥0.2
FDR ≤0.01 and abs ΔPSI <0.2
FDR >0.01
B
Non-subset #2 CLL
SF3B1^WT (n=58)
SF3B1^MUT (n=7)
Non-subset #2 comparison
Subset #2 comparison
Gene
ID
ΔPSI
VAF
PSI
0%
100%
SF3B1 mutation
Subset
p.K666E
p.K700E
p.T663I
p.K700E
p.H662Q
p.K700E
p.K700E
C
Non-subset #2 CLL
ΔPSI=29.1%
P<0.001
ΔPSI=44.6%
P<0.001
ΔPSI=6.6%
P=0.333
ΔPSI=12.1%
P=0.200
ΔPSI=-14.2%
P=0.006
ΔPSI=-30.1%
P=0.003
ΔPSI=-11.6%
P=0.049
ΔPSI=33%
P<0.001
ΔPSI=31.4%
P<0.001
PSI
WT
MUT
ZEB1
BRD9
PLSCR1
TENT4B
CXXC1
DCAF16
UBP1
DLST
SERBP1

Supplementary Figure 1. *Occurrence of significant $SF3B1^{MUT}$ subset #2-related ASEs in other CLL subsets.*

A. Volcano plots for each category of ASEs detected in the comparison of 18 *SF3B1*^{MUT} and 17 *SF3B1*^{WT} subset #2 cases. Red dots indicate ASEs that are considered significant (|ΔPSI| ≥ 20% and FDR ≤ 0.01). **B.** Heatmap illustrating the individual PSI values for *SF3B1*^{MUT} subset #2 -related ASEs in subset #1 (n = 25), subset #3 (n = 5), subset #4 (n = 14), subset #5 (n = 4), subset #6 (n = 4), subset #7 (n = 4), subset #59 (n = 1), subset #99 (n = 4), and subset #169 (n = 4) cases. Of all cases, 7 are *SF3B1*^{MUT} and 55 *SF3B1*^{WT}, respectively. ASEs are displayed based on the ASE category and arranged in the same way as in the subset #2 comparison. The bar plot to the right of the heatmap shows ΔPSI values for the current comparison of *SF3B1*^{MUT} and *SF3B1*^{WT} in dark grey and the subset #2 comparison in light grey. Red asterisks mark ASEs that appeared significant in both comparisons. Of the 80 ASEs identified in the comparison of *SF3B1*^{MUT} and *SF3B1*^{WT} subset #2 cases, 79 were observed in the current comparison, with 28 surpassing the significance threshold. ASE in *MCTP2* was not detected. The gene affected by each ASE and the corresponding unique ASE ID specific to the current comparison are listed. Alternatively spliced transcripts that encode ncBAF complex-interacting proteins are depicted in color. For multiple ASEs per gene, only the top ASE is colored, while the others are marked with black asterisks. **C.** Scattered box plot showing the PSI value distribution, ΔPSI values, and *P* values (Wilcoxon rank-sum test) for significant ncBAF complex-related ASEs (identified in the subset #2 CLL alternative splicing analysis) in other *SF3B1*^{MUT} and *SF3B1*^{WT} subset cases. The specific ASEs for *ZEB1*, *BRD9*, *PLSCR1*, *TENT4B*, *CXXC1*, *DCAF16*, *UBP1*, *DLST*, and *SERBP1* have the unique ASE IDs ZEB1_SE_80841, BRD9_SE_172156, PLSCR1_SE_158106, PAPD5_SE_37399, CXXC1_SE_48540, DCAF16_SE_175422, UBP1_SE_116600, DLST_A3SS_4475, and SERBP1_A3SS_5823, respectively, in Supplementary Table 3C, D. The boxes represent the median and the interquartile range (IQR), while the whiskers extend to 1.5 times the IQR from the first and third quartiles. WT: wildtype; MUT: mutated; PSI: percent spliced in; VAF: variant allele frequency.

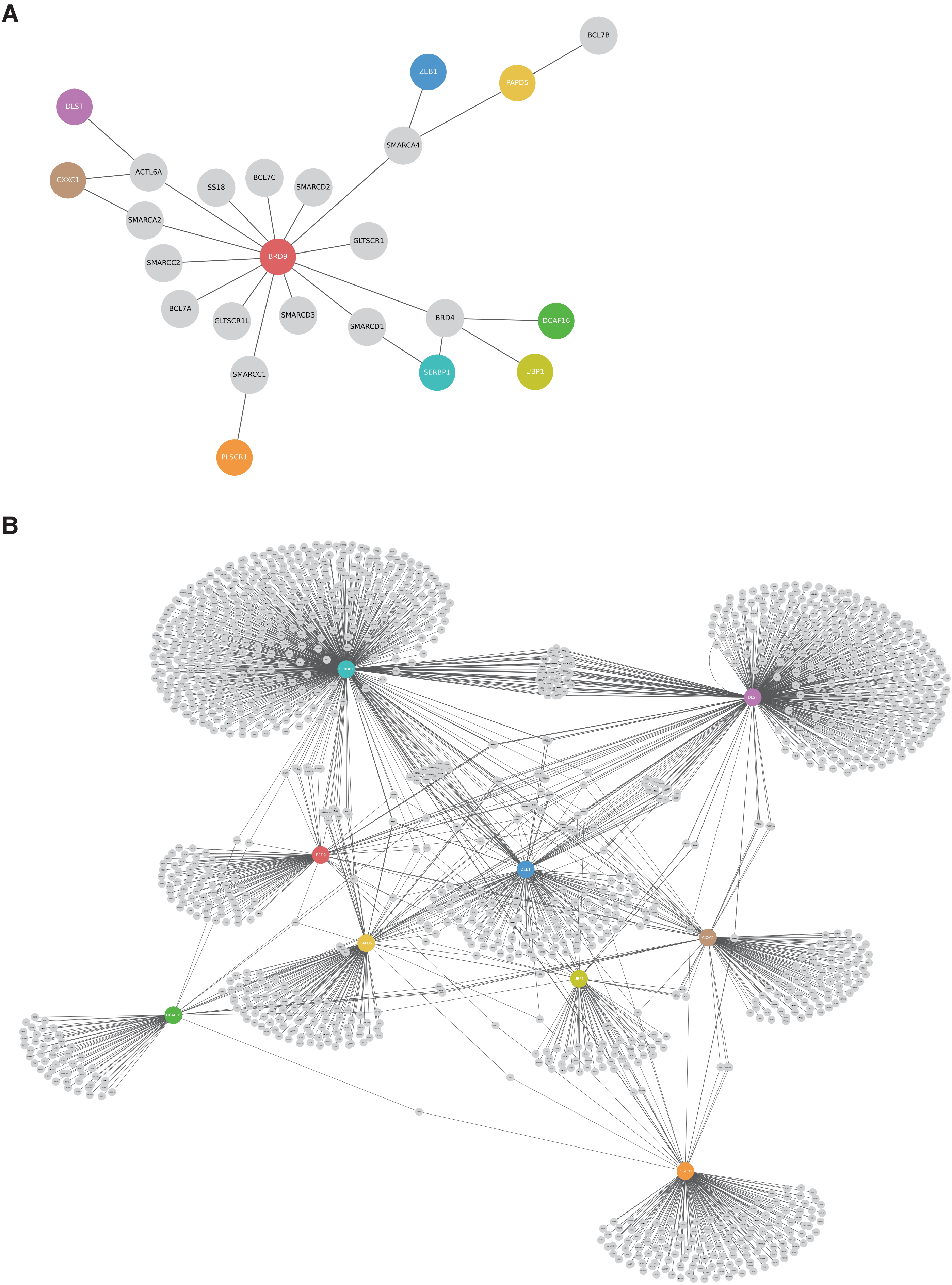
A
BCL7B
ZEB1
PAPD5
DLST
SMARCA4
ACTL6A
CXXC1
SS18
BCL7C
SMARCD2
SMARCA2
GLTSCR1
BRD9
SMARCC2
BCL7A
GLTSCR1L
SMARCD3
SMARCD1
BRD4
DCAF16
SERBP1
UBP1
SMARCC1
PLSCR1
B

Supplementary Figure 2. *Protein-protein interaction network analysis of alternatively spliced transcripts encoding ncBAF complex-related proteins.*

A. Network plot showing protein-protein interactions (PPIs) between ncBAF complex subunits and ZEB1, BRD9, PLSCR1, TENT4B, CXXC1, DCAF16, UBP1, DLST, and SERBP1. All proteins whose genes were found to be affected by alternative splicing in the comparison of $SF3B1^{MUT}$ and $SF3B1^{WT}$ subset #2 cases were queried in BioGRID v4.4[62], but only the aforementioned showed interactions with the ncBAF complex. **B.** Network plot depicting PPIs of the nine proteins with all identified interactors in BioGRID v4.4. The plot showcases both unique and common interactors of these proteins. For all the interactions identified see Supplementary Table 4.

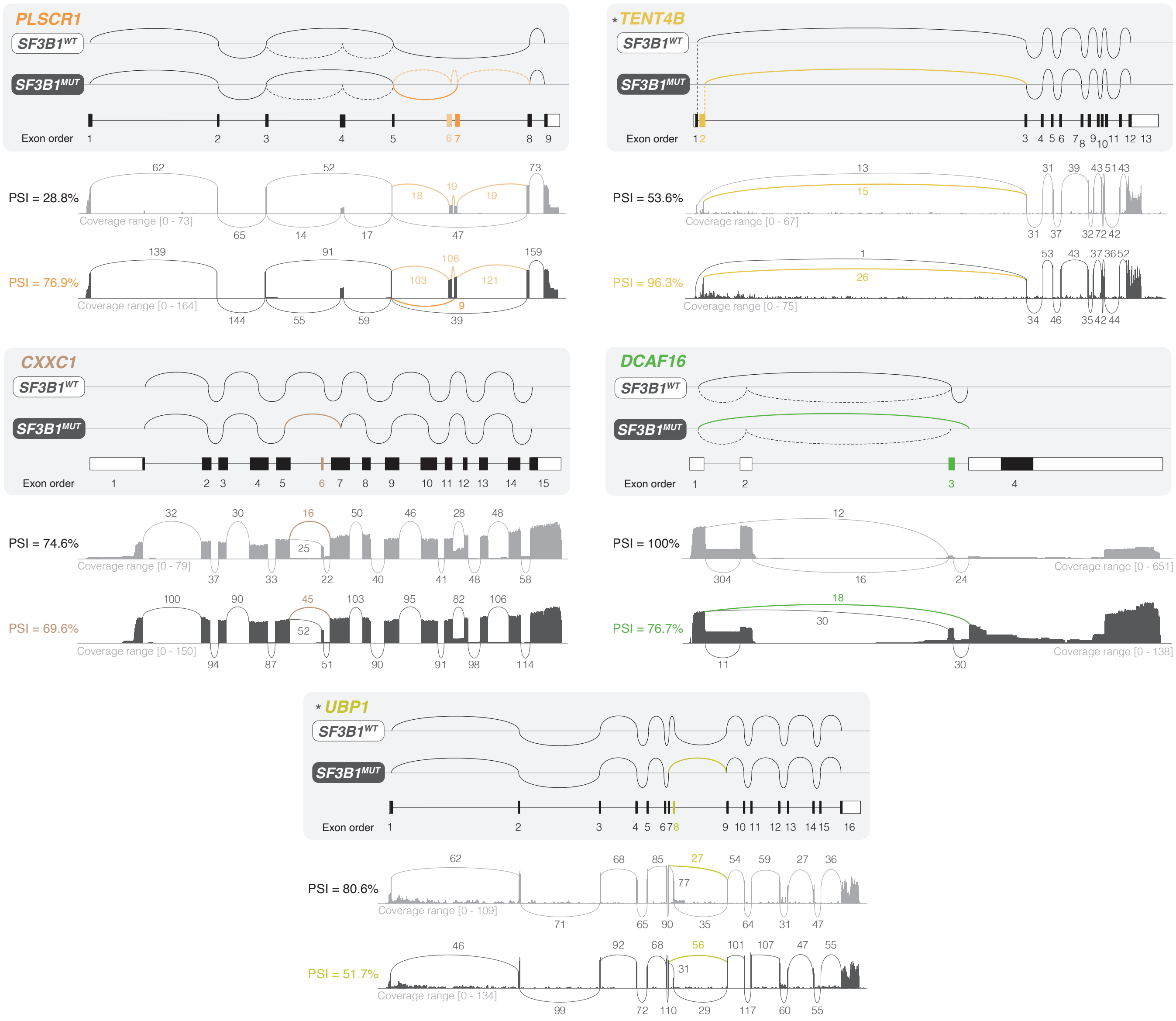

Supplementary Figure 3. *Long-read RNA-sequencing validates the predicted alternative transcripts related to the ncBAF complex in SF3B1^{MUT} CLL.*

Sashimi plots illustrating the identified ASEs and alternative splicing patterns in genes that encode ncBAF complex-interacting proteins in *SF3B1^{WT}* versus *SF3B1^{MUT}* CLL, including *PLSCR1*, *TENT4B*, *CXXC1*, *DCAF16*, and *UBP1*. For each gene, the top two sashimi plots within the gray box illustrate the predicted splice variants in *SF3B1^{WT}* versus *SF3B1^{MUT}* CLL. The colored arc highlights the primary ASE, while lighter arcs represent additional ASEs if present. The gene map indicates the relative location and order of the detected exons in relation to the sequencing results. For each corresponding gene, the lower two sashimi plots show the coverage and splice junction count data from the aligned long-read RNA-seq data from an *SF3B1^{WT}* case (RS24) and an *SF3B1^{MUT}* case (RS55), both belonging to subset #2 CLL. Sufficient read depth was not achieved to validate the full-length *TENT4B* and *UBP1* alternative splice variants, hence short-read RNA-seq data for the same cases is presented. These two genes are denoted with black asterisks. The direction of the genes is arranged from left to right. WT: wildtype; MUT: mutated; PSI: percent spliced in.

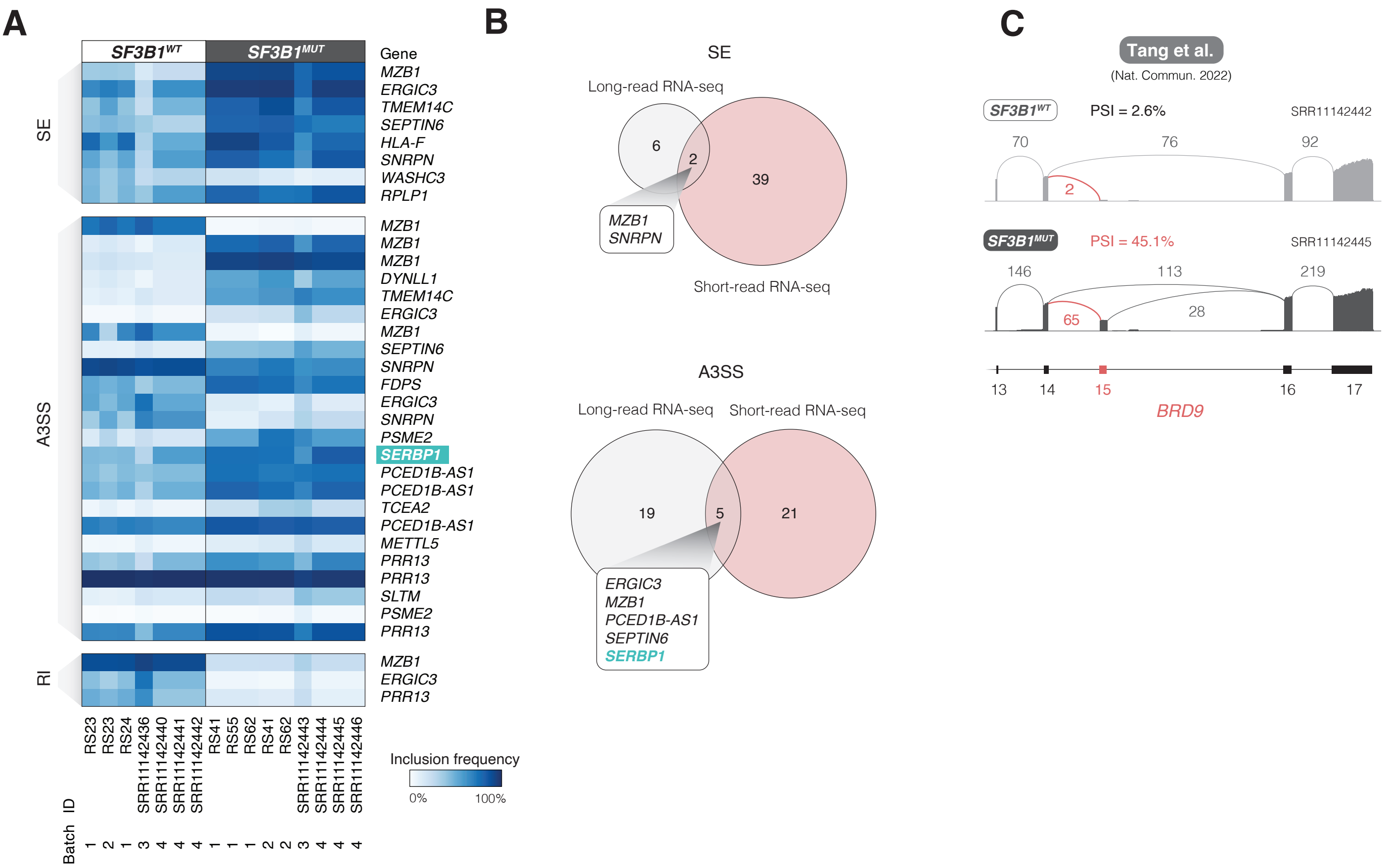

Supplementary Figure 4. *Alternative splicing analysis of long-read RNA-sequencing data from SF3B1^{MUT} and SF3B1^{WT} CLL cases.*

A. Heatmap showing the individual inclusion frequencies for significant ASEs (FDR ≤ 0.05) identified by FLAIR alternative splicing analysis between *SF3B1^{MUT}* and *SF3B1^{WT}* CLL cases from our long-read RNA-seq data and data acquired from Tang et al.[19]. For a detailed view of the alternative splicing analysis results see Supplementary Table 5. **B.** Venn diagrams displaying the overlap of genes in which ASEs were identified by the FLAIR alternative splicing analysis of the long-read RNA-seq data and the rMATS alternative splicing analysis of the subset #2 CLL based on the short-read RNA-seq data. **C.** Sashimi plots based on the long-read RNA-seq data from Tang et al. illustrating the alternatively spliced exon 15 in *BRD9*. The plots depict an *SF3B1^{WT}* case (SRR11142442) and an *SF3B1^{MUT}* case (SRR11142445). WT: wildtype; MUT: mutated; PSI: percent spliced in; SE: skipped exon; A3SS: alternative 3' exon splice site; RI: retained intron.

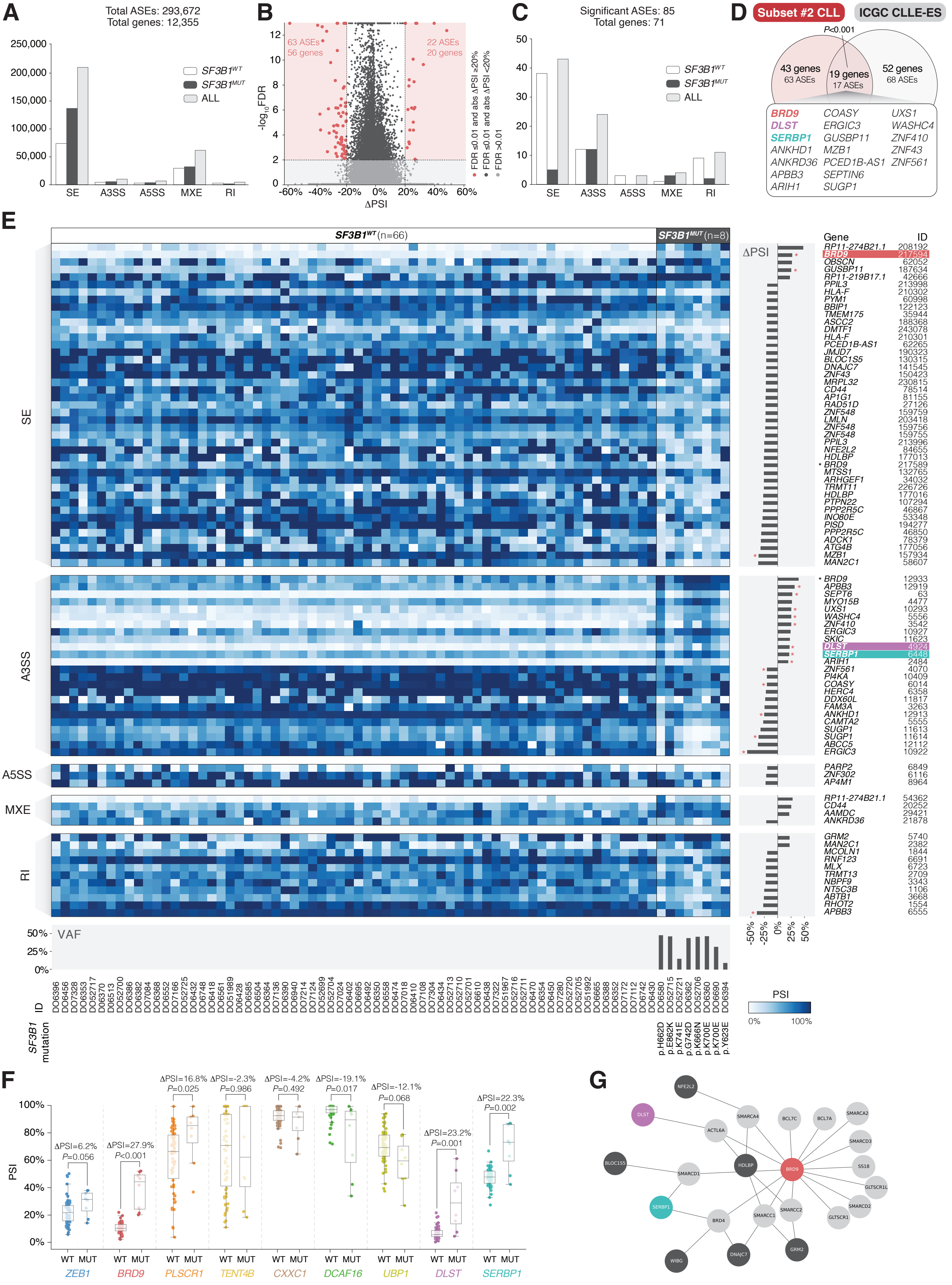
A
Total ASEs: 293,672
Total genes: 12,355
SF3B1WT
SF3B1MUT
ALL
SE A3SS A5SS MXE RI
B
63 ASEs
56 genes
22 ASEs
20 genes
-log10FDR
ΔPSI
FDR ≤0.01 and abs ΔPSI ≥20%
FDR ≤0.01 and abs ΔPSI <20%
FDR >0.01
C
Significant ASEs: 85
Total genes: 71
D
Subset #2 CLL
ICGC CLLE-ES
P<0.001
43 genes
63 ASEs
19 genes
17 ASEs
52 genes
68 ASEs
BRD9 COASY UXS1
DLST ERGIC3 WASHC4
SERBP1 GUSBP11 ZNF410
ANKHD1 MZB1 ZNF43
ANKRD36 PCED1B-AS1 ZNF561
APBB3 SEPTIN6
ARIH1 SUGP1
E
SF3B1WT (n=66)
SF3B1MUT (n=8)
SE
A3SS
A5SS
MXE
RI
ΔPSI
Gene ID
VAF
ID
SF3B1 mutation
p.H662D p.E862K p.K741E p.G742D p.K666N p.K700E p.K700E p.Y623E
PSI
0% 100%
F
PSI
WT MUT
ZEB1 BRD9 PLSCR1 TENT4B CXXC1 DCAF16 UBP1 DLST SERBP1
ΔPSI=6.2% P=0.056
ΔPSI=27.9% P<0.001
ΔPSI=16.8% P=0.025
ΔPSI=-2.3% P=0.986
ΔPSI=-4.2% P=0.492
ΔPSI=-19.1% P=0.017
ΔPSI=-12.1% P=0.068
ΔPSI=23.2% P=0.001
ΔPSI=22.3% P=0.002
G
NFE2L2 DLST SMARCA4 ACTL6A BCL7C BCL7A SMARCA2 SMARCD3 BLOC1S5 SMARCD1 HDLBP BRD9 SS18 GLTSCR1L SERBP1 BRD4 SMARCC1 SMARCC2 SMARCD2 GLTSCR1 WIBG DNAJC7 GRM2

Supplementary Figure 5*. Alternative splicing analysis of ICGC CLLE-ES.*

A. Bar plot displaying numbers of all identified ASEs in the comparison of 8 *SF3B1*^{MUT} and 66 *SF3B1*^{WT} cases in the ICGC CLLE-ES dataset[30,31] across different ASE categories. 293,672 ASEs were detected and involved transcripts of 12,355 genes. **B.** Volcano plot depicting all identified ASEs in the comparison of *SF3B1*^{MUT} and *SF3B1*^{WT} ICGC CLLE-ES cases. Red dots indicate ASEs that are considered significant ($|\Delta PSI| \geq 20\%$ and $FDR \leq 0.01$; 85 ASEs). **C.** Bar plot displaying numbers of significant ASEs in the comparison of *SF3B1*^{MUT} and *SF3B1*^{WT} ICGC CLLE-ES cases across different ASE categories. 85 ASEs were considered significant and involved transcripts of 71 genes. **D.** Venn diagram illustrating genes affected by significant *SF3B1*^{MUT}-related ASEs identified in subset #2 cases and ICGC CLLE-ES cases. The 19 genes found to be affected by alternative splicing in two independent datasets are listed. Remarkably, 17 identical ASEs were determined to be statistically significant in two independent datasets (binomial test). **E.** Heatmap illustrating the individual PSI values for the 85 significant ASEs detected in the comparison of *SF3B1*^{MUT} and *SF3B1*^{WT} ICGC CLLE-ES cases. 26 ASEs occurred within the same gene at least twice. ASEs are displayed based on the ASE category. The bar plot to the right of the heatmap shows ΔPSI values. Red asterisks mark ASEs that appeared significant both in the ICGC CLLE-ES and subset #2 comparison. The gene affected by each ASE and the corresponding unique ASE ID specific to the current comparison are listed. Alternatively spliced transcripts that encode ncBAF complex-interacting proteins are depicted in color. For multiple ASEs per gene, only the top ASE is colored, while the others are marked with black asterisks. **F.** Scattered box plot showing the distribution of PSI values, ΔPSI values, and *P* values (Wilcoxon rank-sum test) for significant ncBAF complex-related ASEs (identified in the subset #2 CLL alternative splicing analysis) in *SF3B1*^{MUT} and *SF3B1*^{WT} ICGC CLLE-ES cases. The specific ASEs for *ZEB1*, *BRD9*, *PLSCR1*, *TENT4B*, *CXXC1*, *DCAF16*, *UBP1*, *DLST*, and *SERBP1* have the unique ASE IDs ZEB1_SE_102203_ICGC, BRD9_SE_217594_ICGC, PLSCR1_SE_200333_ICGC, PAPD5_SE_47549_ICGC, CXXC1_SE_221667_ICGC, DCAF16_SE_61446_ICGC, UBP1_SE_148372_ICGC, DLST_A3SS_4924_ICGC, and SERBP1_A3SS_6448_ICGC, respectively, in Supplementary Table 6A, B. The boxes represent the median and the IQR, while the whiskers extend to 1.5 times the IQR from the first and third quartiles. **G.** Network plot showing PPIs between ncBAF complex subunits and significant ncBAF complex-related ASEs identified in the ICGC CLLE-ES alternative splicing analysis, BLOC1S5, BRD9, DLST, DNAJC7, GRM2, HDLBP, NFE2L2, SERBP1, and WIBG. All proteins whose genes are affected by alternative splicing were queried in BioGRID v4.4[62], but only the aforementioned showed interactions with the ncBAF complex. ASE: alternative splicing event; WT: wildtype; MUT: mutated; PSI: percent spliced in; VAF: variant allele frequency.

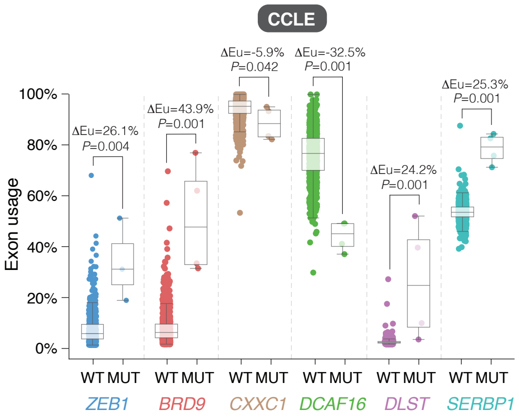

Supplementary Figure 6. *Alternative splicing analysis of CCLE cell lines.*

Scattered box plot showing the distribution of exon usage (Eu) values, ΔEu values, and *P* values (Wilcoxon rank-sum test) for significant ncBAF complex-related ASEs in the comparison between 4 $SF3B1^{MUT}$ and 1,015 $SF3B1^{WT}$ CCLE cell lines[40]. The specific ASEs for *ZEB1*, *BRD9*, *CXXC1*, *DCAF16*, *DLST*, and *SERBP1* have the unique ASE IDs chr10_31661947_31662102_ZEB1_5p, chr5_869519_869360_BRD9_5p, chr18_47811721_47811695_CXXC1_5p, chr4_17806848_17806730_DCAF16_3p, chr14_75356581_75356655_DLST_5p, and chr1_67890660_67890571_SERBP1_5p, respectively (file 'CCLE_RNAseq_ExonUsageRatio_20180929.gct' from DepMap). The boxes represent the median and the IQR, while the whiskers extend to 1.5 times the IQR from the first and third quartiles. WT: wildtype; MUT: mutated; Eu: exon usage.

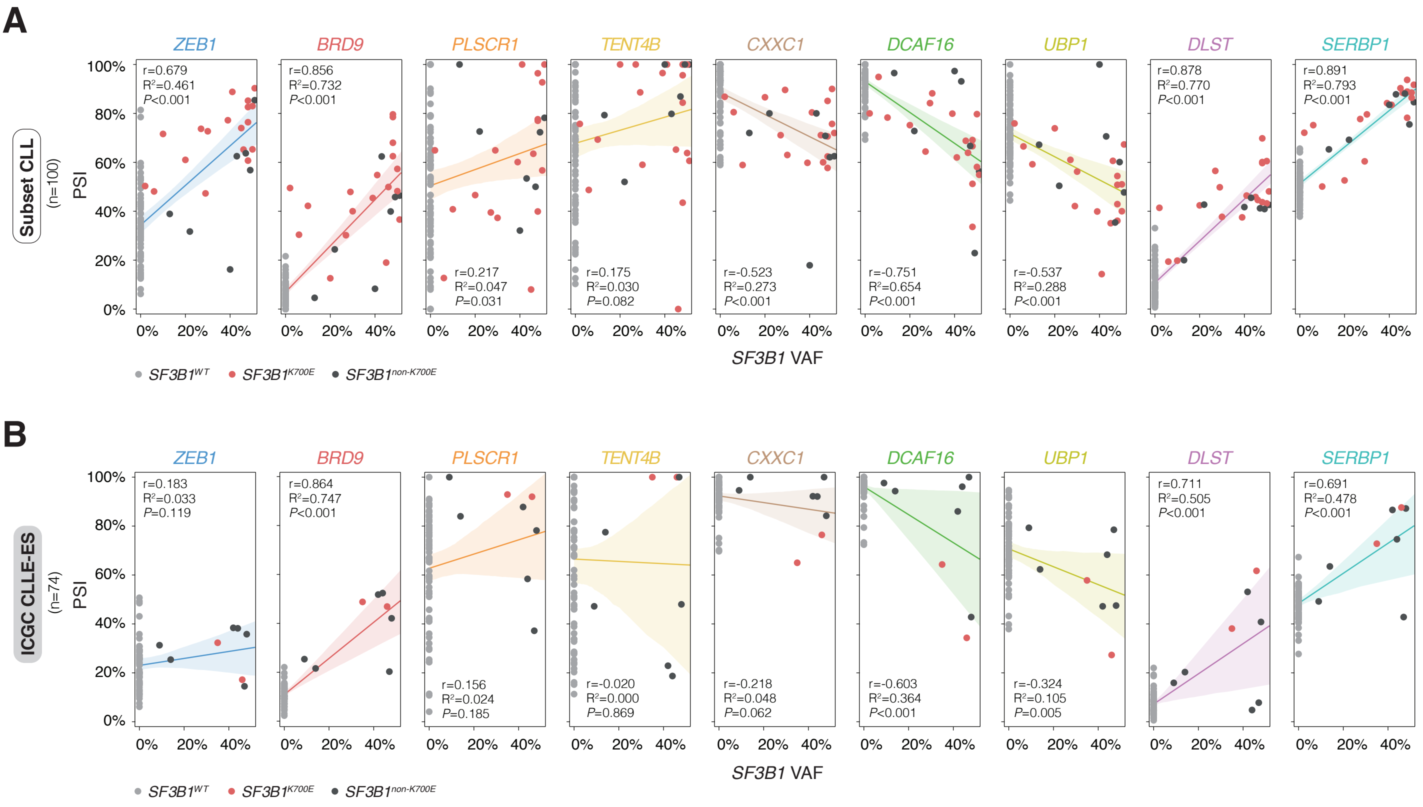

***Supplementary Figure 7.** Correlation of PSI values and SF3B1 variant allele frequencies for ncBAF complex-related ASEs in subset CLL and ICGC CLLE-ES.*

A. Pearson correlation and linear regression model fit between individual PSI values of the significant ncBAF complex-related ASEs (identified in the subset #2 CLL alternative splicing analysis) and RNA-seq-based *SF3B1* VAFs within 100 subset cases. Liner regression model fits are shown with 95% confidence intervals. For *SF3B1* mutation status see Supplementary Table 1A. **B.** Pearson correlation and linear regression model fit as in panel A but for 74 ICGC CLLE-ES cases[33,34]. For *SF3B1* mutation status see Supplementary Table 1B. WT: wildtype; MUT: mutated; PSI: percent spliced in; VAF: variant allele frequency.

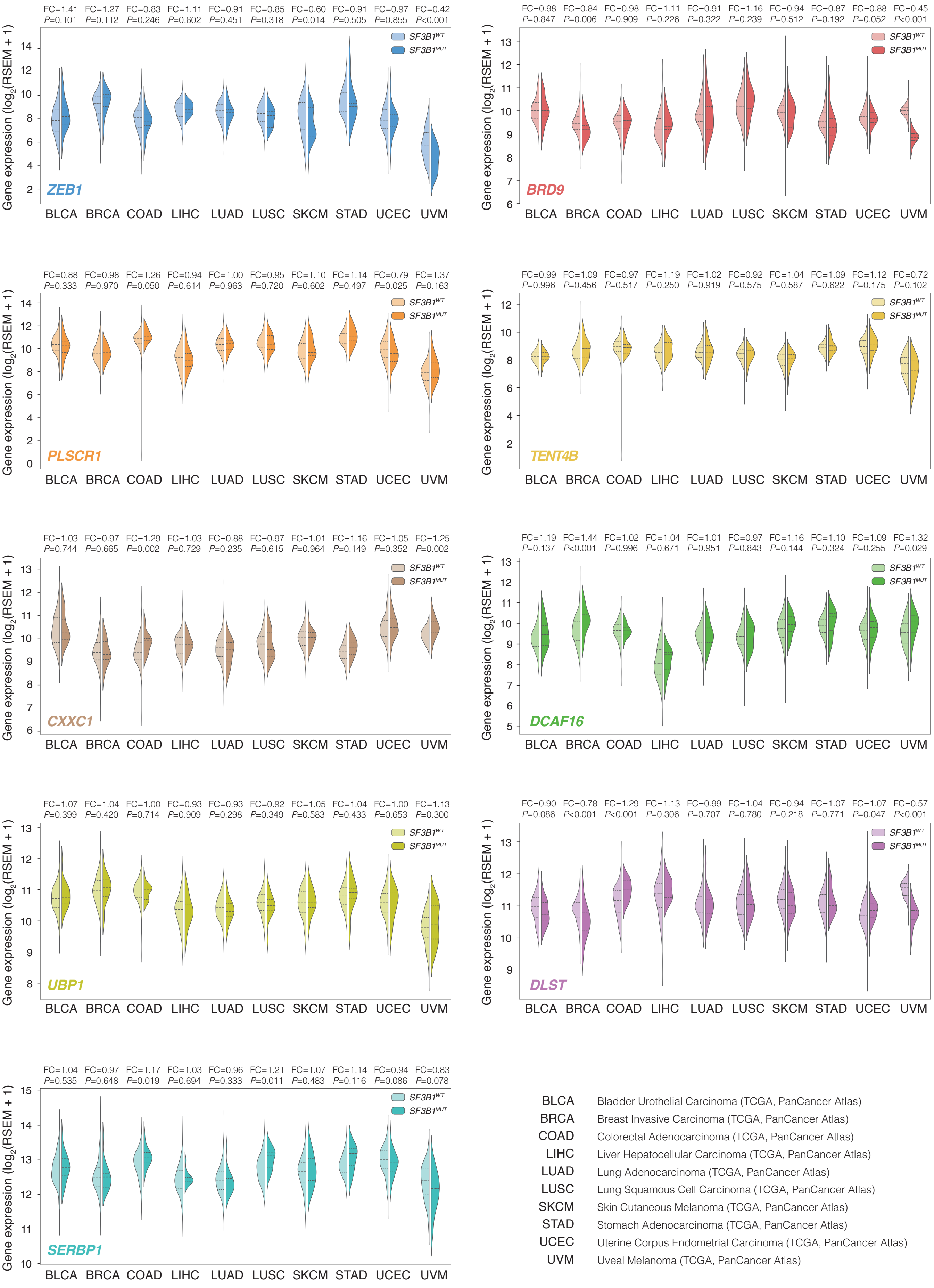
ZEB1
FC=1.41 P=0.101; FC=1.27 P=0.112; FC=0.83 P=0.246; FC=1.11 P=0.602; FC=0.91 P=0.451; FC=0.85 P=0.318; FC=0.60 P=0.014; FC=0.91 P=0.505; FC=0.97 P=0.855; FC=0.42 P<0.001
BRD9
FC=0.98 P=0.847; FC=0.84 P=0.006; FC=0.98 P=0.909; FC=1.11 P=0.226; FC=0.91 P=0.322; FC=1.16 P=0.239; FC=0.94 P=0.512; FC=0.87 P=0.192; FC=0.88 P=0.052; FC=0.45 P<0.001
PLSCR1
FC=0.88 P=0.333; FC=0.98 P=0.970; FC=1.26 P=0.050; FC=0.94 P=0.614; FC=1.00 P=0.963; FC=0.95 P=0.720; FC=1.10 P=0.602; FC=1.14 P=0.497; FC=0.79 P=0.025; FC=1.37 P=0.163
TENT4B
FC=0.99 P=0.996; FC=1.09 P=0.456; FC=0.97 P=0.517; FC=1.19 P=0.250; FC=1.02 P=0.919; FC=0.92 P=0.575; FC=1.04 P=0.587; FC=1.09 P=0.622; FC=1.12 P=0.175; FC=0.72 P=0.102
CXXC1
FC=1.03 P=0.744; FC=0.97 P=0.665; FC=1.29 P=0.002; FC=1.03 P=0.729; FC=0.88 P=0.235; FC=0.97 P=0.615; FC=1.01 P=0.964; FC=1.16 P=0.149; FC=1.05 P=0.352; FC=1.25 P=0.002
DCAF16
FC=1.19 P=0.137; FC=1.44 P<0.001; FC=1.02 P=0.996; FC=1.04 P=0.671; FC=1.01 P=0.951; FC=0.97 P=0.843; FC=1.16 P=0.144; FC=1.10 P=0.324; FC=1.09 P=0.255; FC=1.32 P=0.029
UBP1
FC=1.07 P=0.399; FC=1.04 P=0.420; FC=1.00 P=0.714; FC=0.93 P=0.909; FC=0.93 P=0.298; FC=0.92 P=0.349; FC=1.05 P=0.583; FC=1.04 P=0.433; FC=1.00 P=0.653; FC=1.13 P=0.300
DLST
FC=0.90 P=0.086; FC=0.78 P<0.001; FC=1.29 P<0.001; FC=1.13 P=0.306; FC=0.99 P=0.707; FC=1.04 P=0.780; FC=0.94 P=0.218; FC=1.07 P=0.771; FC=1.07 P=0.047; FC=0.57 P<0.001
SERBP1
FC=1.04 P=0.535; FC=0.97 P=0.648; FC=1.17 P=0.019; FC=1.03 P=0.694; FC=0.96 P=0.333; FC=1.21 P=0.011; FC=1.07 P=0.483; FC=1.14 P=0.116; FC=0.94 P=0.086; FC=0.83 P=0.078
SF3B1^WT
SF3B1^MUT
Gene expression (log2(RSEM + 1)
BLCA BRCA COAD LIHC LUAD LUSC SKCM STAD UCEC UVM
BLCA Bladder Urothelial Carcinoma (TCGA, PanCancer Atlas)
BRCA Breast Invasive Carcinoma (TCGA, PanCancer Atlas)
COAD Colorectal Adenocarcinoma (TCGA, PanCancer Atlas)
LIHC Liver Hepatocellular Carcinoma (TCGA, PanCancer Atlas)
LUAD Lung Adenocarcinoma (TCGA, PanCancer Atlas)
LUSC Lung Squamous Cell Carcinoma (TCGA, PanCancer Atlas)
SKCM Skin Cutaneous Melanoma (TCGA, PanCancer Atlas)
STAD Stomach Adenocarcinoma (TCGA, PanCancer Atlas)
UCEC Uterine Corpus Endometrial Carcinoma (TCGA, PanCancer Atlas)
UVM Uveal Melanoma (TCGA, PanCancer Atlas)

Supplementary Figure 8. *Expression levels of alternatively spliced ncBAF complex-related genes in relation to SF3B1 mutation status across various cancer types.*

Violin plots displaying relative expression levels of alternatively spliced ncBAF complex-related genes in different cancer types from TCGA with reported *SF3B1* mutations[35–38]. The fold differences and *P* values (Wilcoxon rank-sum test) of comparisons between $SF3B1^{MUT}$ and $SF3B1^{WT}$ cases for each cancer type are indicated. For a detailed view of gene expression values see Supplementary Table 7A, B. WT: wildtype; MUT: mutated; FC: fold change.

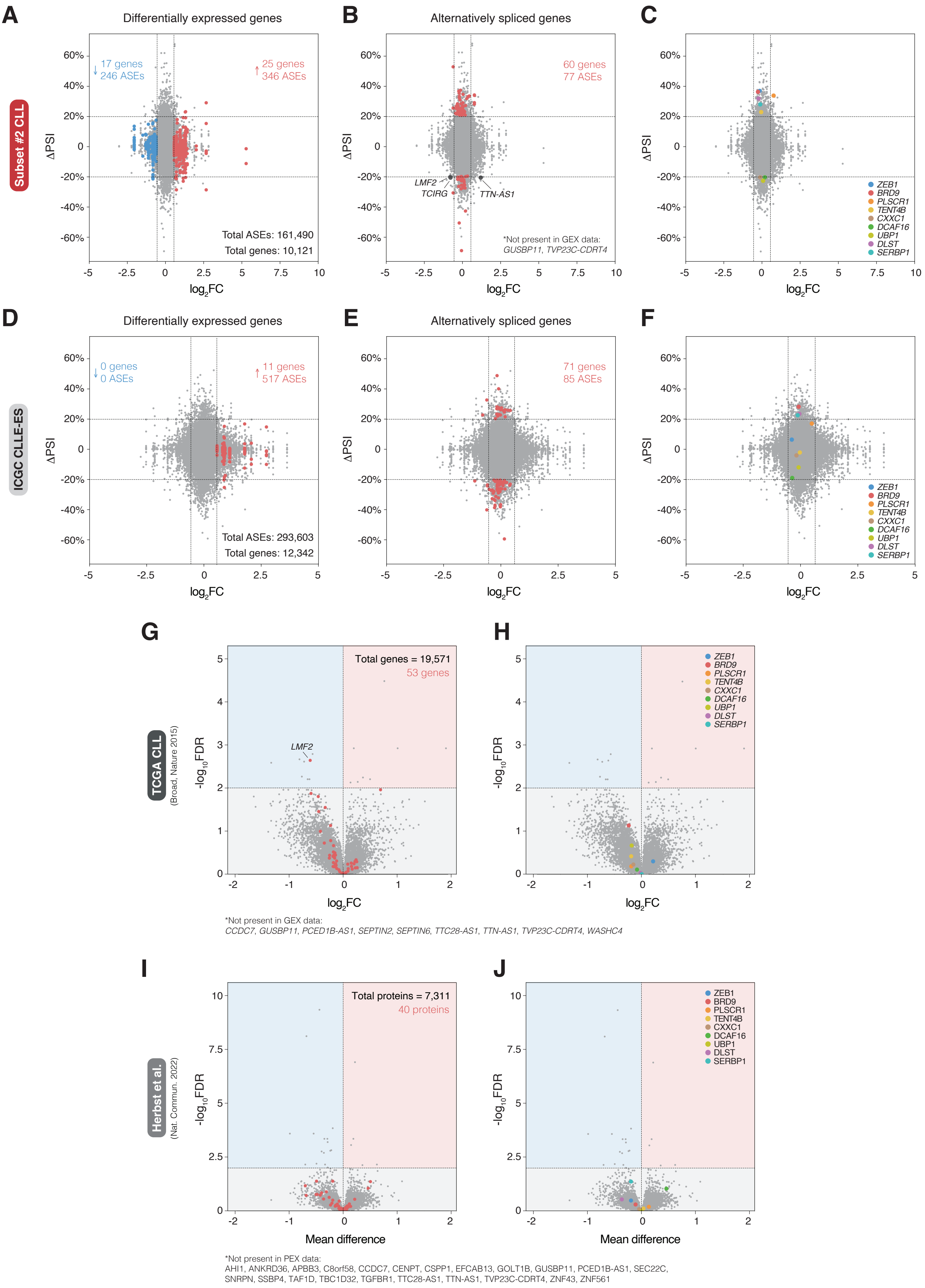
A
Differentially expressed genes
B
Alternatively spliced genes
C
Subset #2 CLL
ΔPSI
↓ 17 genes
246 ASEs
↑ 25 genes
346 ASEs
Total ASEs: 161,490
Total genes: 10,121
60 genes
77 ASEs
LMF2
TCIRG
TTN-AS1
*Not present in GEX data:
GUSBP11, TVP23C-CDRT4
ZEB1
BRD9
PLSCR1
TENT4B
CXXC1
DCAF16
UBP1
DLST
SERBP1
log2FC
D
Differentially expressed genes
E
Alternatively spliced genes
F
ICGC CLLE-ES
↓ 0 genes
0 ASEs
↑ 11 genes
517 ASEs
Total ASEs: 293,603
Total genes: 12,342
71 genes
85 ASEs
G
H
TCGA CLL
(Broad, Nature 2015)
-log10FDR
Total genes = 19,571
53 genes
LMF2
log2FC
*Not present in GEX data:
CCDC7, GUSBP11, PCED1B-AS1, SEPTIN2, SEPTIN6, TTC28-AS1, TTN-AS1, TVP23C-CDRT4, WASHC4
I
J
Herbst et al.
(Nat. Commun. 2022)
Total proteins = 7,311
40 proteins
Mean difference
*Not present in PEX data:
AHI1, ANKRD36, APBB3, C8orf58, CCDC7, CENPT, CSPP1, EFCAB13, GOLT1B, GUSBP11, PCED1B-AS1, SEC22C,
SNRPN, SSBP4, TAF1D, TBC1D32, TGFBR1, TTC28-AS1, TTN-AS1, TVP23C-CDRT4, ZNF43, ZNF561

Supplementary Figure 9. *Gene and protein expression patterns of genes that undergo alternative splicing in $SF3B1^{MUT}$ CLL in different CLL datasets.*

A. Scatter plot depicting individual ASEs identified in the subset #2 CLL alternative splicing analysis and their corresponding ΔPSI values matched to their gene-based $\log_2$FC values from the differential gene expression analysis. Significantly upregulated genes in the comparison of $SF3B1^{MUT}$ and $SF3B1^{WT}$ subset #2 cases (25 genes with 346 ASEs; $\log_2FC \geq 0.58$ and FDR < 0.01) are highlighted in red, while those downregulated (17 genes with 246 ASEs; $\log_2FC \leq -0.58$ and FDR < 0.01) are depicted in blue. A total of 10,121 genes (63.19% of all genes included in the differential gene expression analysis) with 161,490 ASEs (99.01% of all ASEs identified in the alternative splicing analysis) were matched with gene expression data. No ASEs were identified in 5 downregulated and 30 upregulated genes that were found to be differentially expressed. **B.** Scatter plot showing the same relationship as in panel A but highlighting significant ASEs identified in the subset #2 CLL alternative splicing analysis (77 ASEs in 60 genes; |ΔPSI| ≥ 20% and FDR ≤ 0.01) in red. *LMF2*, *TCIRG*, and *TTN-AS1* were found to be both alternatively spliced and differentially expressed. No gene expression data was available for 2 genes with 3 ASEs, namely *GUSBP11* and *TVP23C-CDRT4*. **C.** Scatter plot showing the same relationship as in panels A and B but depicting the significant ncBAF complex-related ASEs identified in the subset #2 CLL alternative splicing analysis. **D.** Scatter plot depicting individual ASEs identified in the ICGC CLLE-ES[33,34] alternative splicing analysis and their corresponding ΔPSI values matched to their gene-based $\log_2$FC values from the differential gene expression analysis. Significantly upregulated genes in the comparison of $SF3B1^{MUT}$ and $SF3B1^{WT}$ ICGC CLLE-ES cases (11 genes with 517 ASEs; $\log_2FC \geq 0.58$ and FDR < 0.01) are highlighted in red. A total of 12,342 genes (49.17% of all genes included in the differential gene expression analysis) with 293,603 ASEs (99.98% of all ASEs identified in the alternative splicing analysis) were matched with gene expression data. No ASEs were identified in 5 downregulated and 6 upregulated genes that were found to be differentially expressed. **E.** Scatter plot showing the same relationship as in panel D but highlighting significant ASEs identified in the ICGC CLLE-ES alternative splicing analysis (85 ASEs in 71 genes; |ΔPSI| ≥ 20% and FDR ≤ 0.01) in red. No genes were found to be both alternatively spliced and differentially expressed. **F.** Scatter plot showing the same relationship as in panels D and E but depicting the significant ncBAF complex-related ASEs (identified in the subset #2 CLL alternative splicing analysis) in ICGC CLLE-ES dataset. **G.** Volcano plot showing differentially expressed genes in the TCGA CLL dataset[35,37,38]. Significant alternatively spliced transcripts (|ΔPSI| ≥ 20% and FDR ≤ 0.01) identified in the subset #2 CLL alternative splicing analysis are highlighted in red. *LMF2* was found to be both alternatively spliced and differentially expressed. No gene expression data was available for 9 genes, namely *CCDC7*, *GUSBP11*, *PCED1B-AS1*, *SEPTIN2*, *SEPTIN6*, *TTC28-AS1*, *TTN-AS1*, *TVP23C-CDRT4*, and *WASHC4*. **H.** Volcano plot showing the same differentially expressed genes as in panel G but highlighting the significant alternatively spliced ncBAF complex-related genes (identified in the subset #2 CLL alternative splicing analysis) in the TCGA CLL dataset. **I.** Volcano plot showing differentially expressed proteins in the Herbst et al. dataset[45]. Corresponding proteins of the significant alternatively spliced transcripts (|ΔPSI| ≥ 20% and FDR ≤ 0.01) identified in the subset #2 CLL alternative splicing analysis are highlighted in red. None of them were found

to be differentially expressed. **J.** Volcano plot showing the same differentially expressed proteins as in panel I but highlighting the significant ncBAF complex-related proteins, whose genes undergo alternative splicing as identified in the subset #2 CLL alternative splicing analysis, in the Herbst et al. dataset. ASE: alternative splicing event; PSI: percent spliced in; FC: fold change; GEX: gene expression; PEX: protein expression.

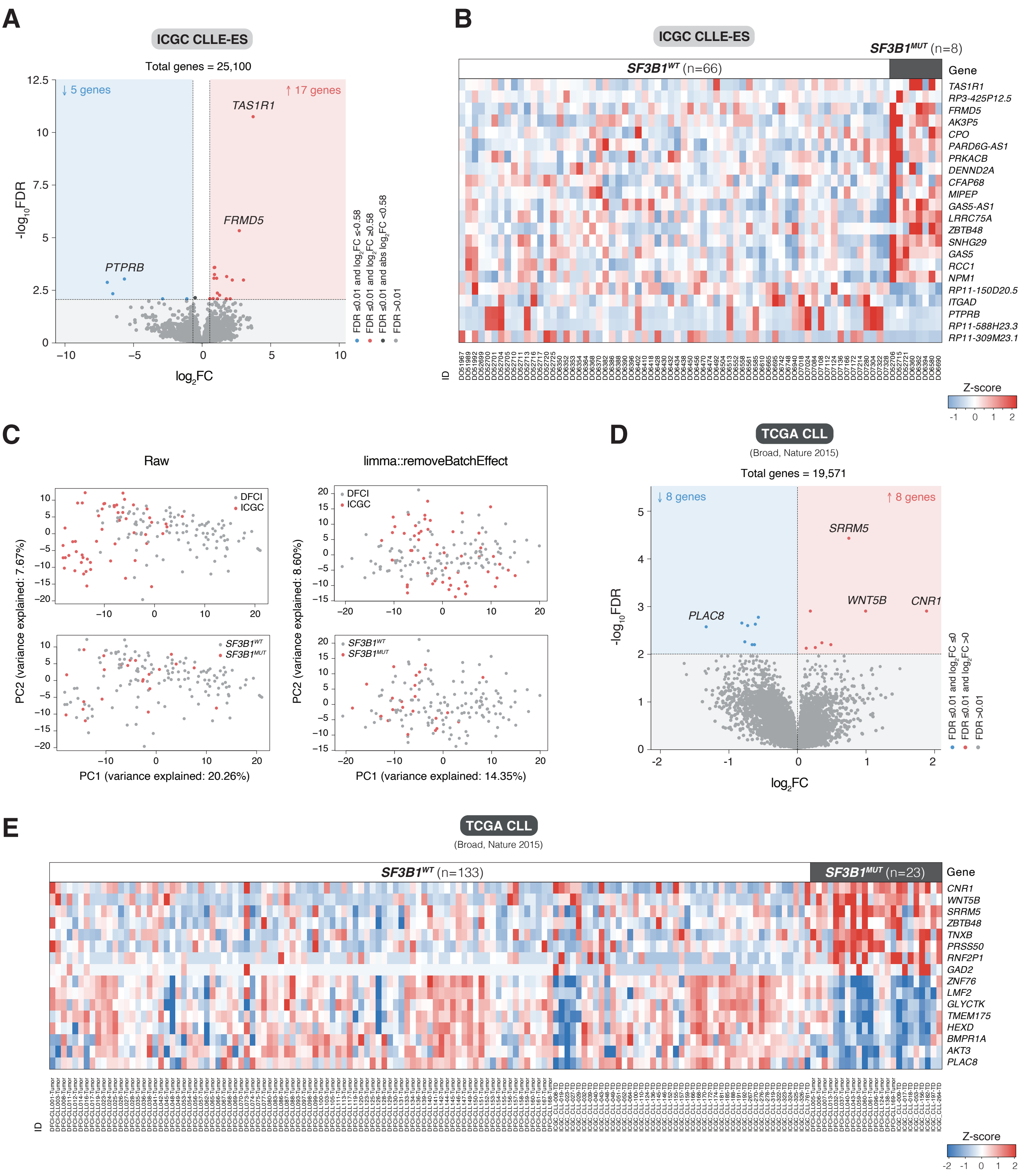

***Supplementary Figure 10**. Differential gene expression analysis between SF3B1^{MUT} and SF3B1^{WT} CLL cases in ICGC CLLE-ES and TGCA CLL.*

A. Volcano plot depicting differentially expressed genes between 8 *SF3B1*^{MUT} and 66 *SF3B1*^{WT} cases in the ICGC CLLE-ES dataset[33,34] with 5 downregulated and 17 upregulated genes ($|\log_2 FC| \geq 0.58$ and $FDR < 0.01$). **B.** Heatmap illustrating *SF3B1*^{MUT} and *SF3B1*^{WT} ICGC CLLE-ES cases based on differential gene expression. **C.** PCA plots depicting the relationship

between cases in the TCGA CLL dataset[35,37,38] based on their gene expression profiles before and after batch effect correction. The upper plots are color-coded based on the batch, while the lower plots are colored according to the *SF3B1* mutation status. **D.** Volcano plot depicting differentially expressed genes between 23 *SF3B1*^{MUT} and 133 *SF3B1*^{WT} TCGA CLL cases with 8 downregulated and 8 upregulated genes (FDR $<$ 0.01). **E.** Heatmap illustrating *SF3B1*^{MUT} and *SF3B1*^{WT} TCGA CLL cases based on differential gene expression. WT: wildtype; MUT: mutated; FDR: false discovery rate; FC: fold change; PC: principal component.

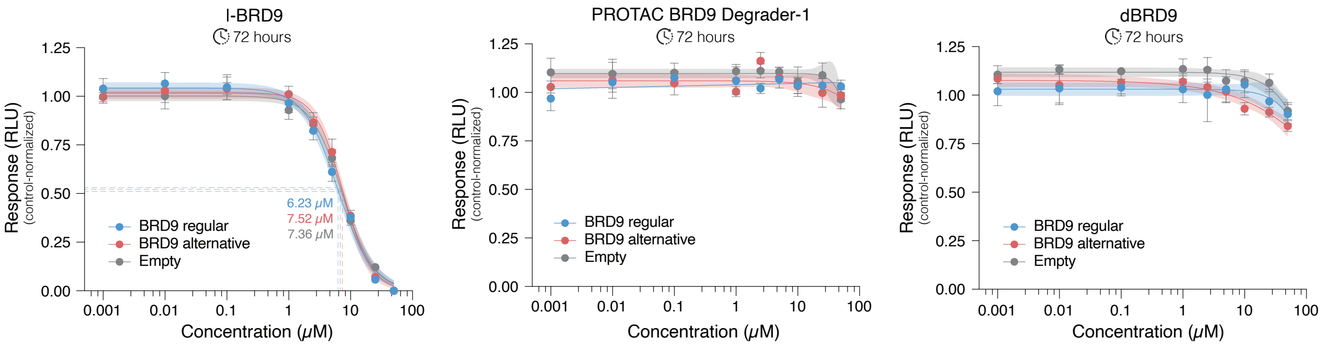

Supplementary Figure 11. *BRD9 inhibition in HEK293T cell lines stably overexpressing regular and alternative BRD9 isoforms.*

Dose-response analysis of I-BRD9, PROTAC BRD9 Degrader-1, and dBRD9 treatments in HEK293T cell lines stably overexpressing regular and alternative BRD9 isoforms. A control cell line transduced with the empty viral backbone was used as a control. The cell lines were treated with drug concentrations ranging from 0.001 to 50 µM for 3 days, and cell viability was determined by CellTiter-Glo 2.0. Complete cell killing was exclusively observed with I-BRD9 treatment, allowing for the determination of corresponding IC_{50} values for each cell line. Dose-response curves are shown with 95% confidence intervals, while individual dots display the mean values of triplicates, with error bars representing the standard deviation. RLU: relative luminescence unit.

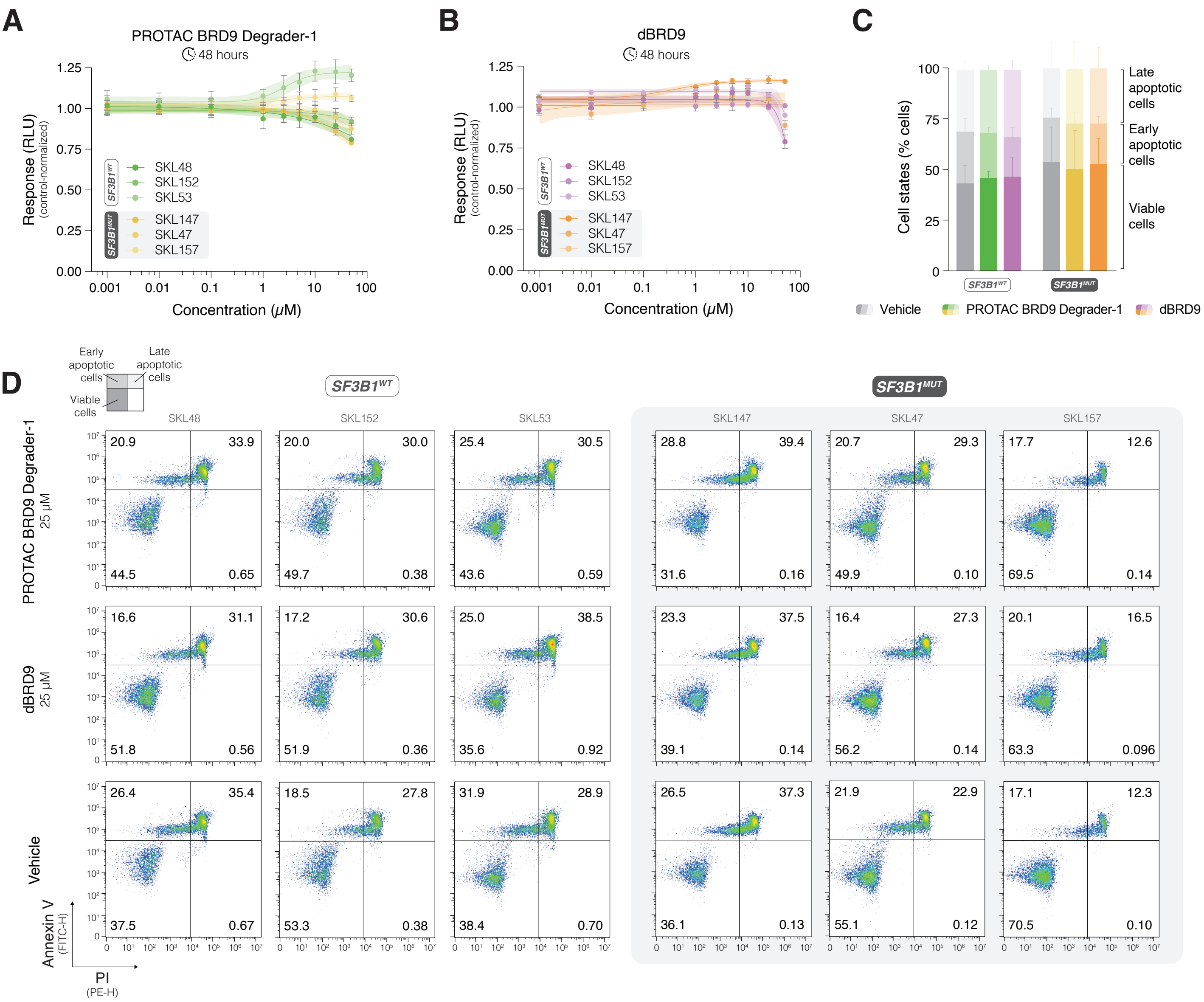

Supplementary Figure 12. *BRD9 inhibition in primary CLL cells.*

A. Dose-response analysis of PROTAC BRD9 Degrader-1 treatment in 3 *SF3B1^WT^* (SKL48, SKL152, SKL53) and 3 *SF3B1^MUT^* (SKL147, SKL47, SKL157) primary CLL cell samples. The cells were treated with drug concentrations ranging from 0.001 to 50 µM for 2 days, and cell viability was determined by CellTiter-Glo 2.0. Dose-response curves are shown with 95% confidence intervals, while individual dots display the mean values of triplicates, with error bars representing the standard deviation. **B.** Dose-response analysis in the same primary CLL cell samples as in panel A but for dBRD9. **C.** Stacked bar plot displaying the summary of apoptosis assessment in the same primary CLL cell samples as in panels A and B. Vehicle (DMSO) was used as the negative control for drug treatment. Differences in the percentages of viable, early apoptotic, and late apoptotic cells compared to negative controls are shown. The bar plots display the mean values, with error bars representing the standard deviation. **D.** Stacked density plots showing apoptosis assessment in the same primary CLL cell samples as in panels A and B, for which summary is shown in panel C. Treatment with 5 µM Camptothecin and vehicle (DMSO) were used as the positive and negative controls, respectively. Apoptosis

was evaluated by Annexin V/PI staining and subsequent flow cytometry analysis. The lower left (Annexin V-/PI- cells), upper left (Annexin V+/PI- cells), and upper right (Annexin V+/PI+ cells) quadrants in the density plots represent the percentages of viable, early apoptotic, and late apoptotic cells, respectively. WT: wildtype; MUT: mutated; RLU: relative luminescence unit.

A

Figure 3E and G

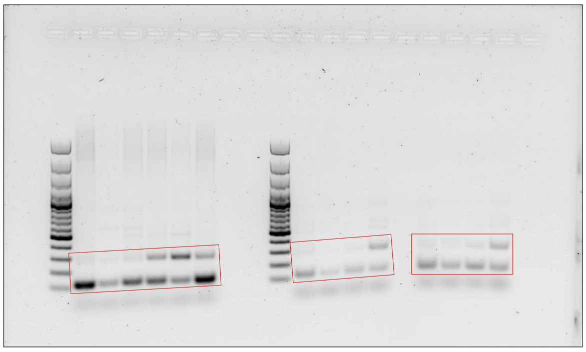

Figure 3I

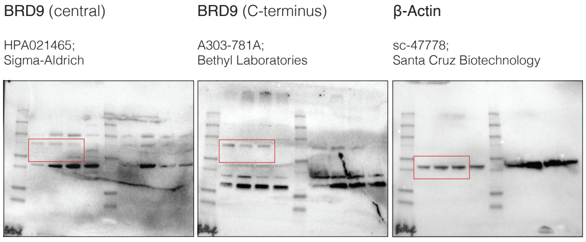

Figure 4F

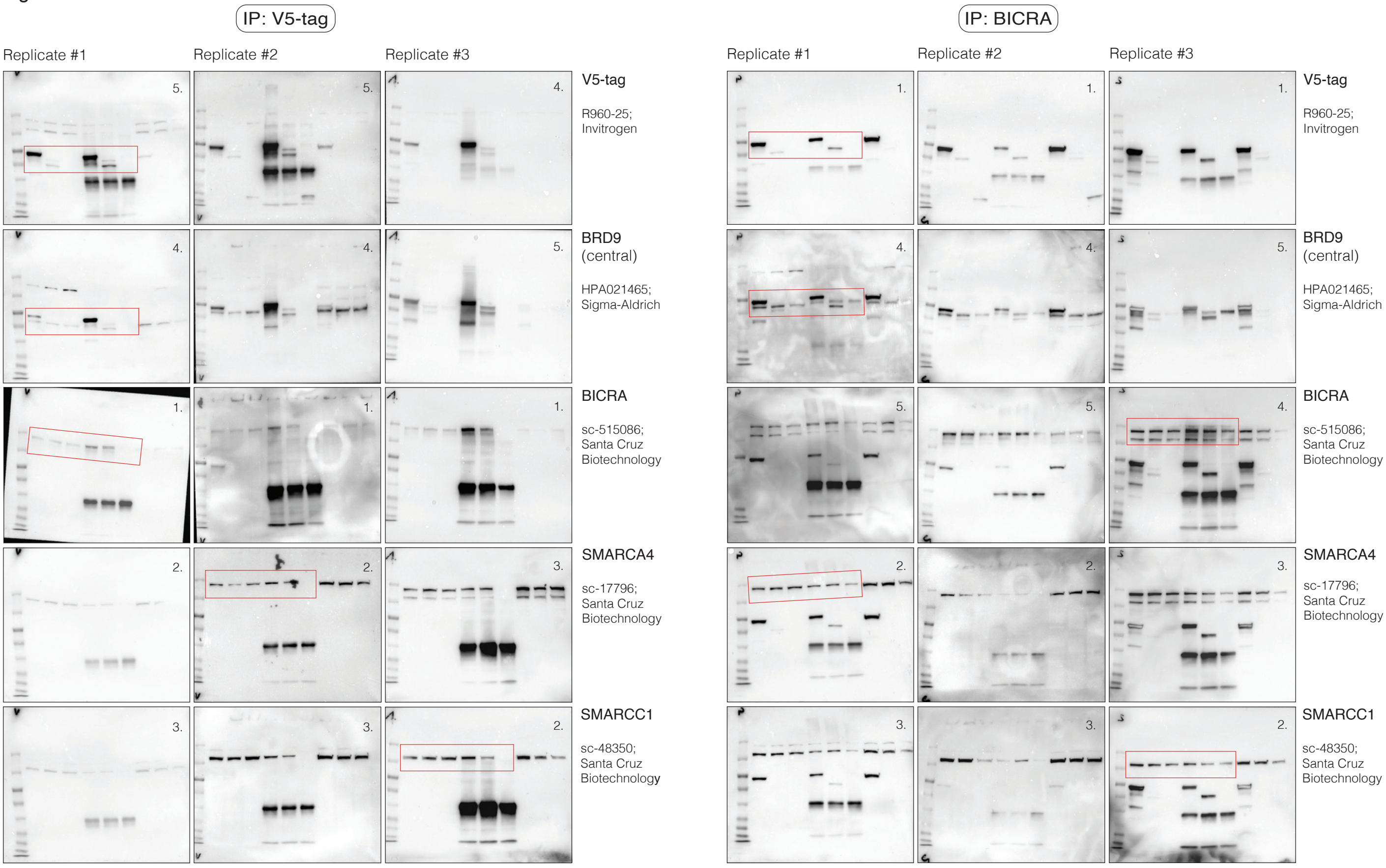

B

Figure 4F and G

IP: V5-tag

Replicate #1		Replicate #2		Replicate #3		
BRD9 regular	BRD9 alternative	BRD9 regular	BRD9 alternative	BRD9 regular	BRD9 alternative	
31912	5407	43079	2258	44709	1437	V5-tag
36686	25160	21362	6241	26070	9018	BICRA
8943	9812	9321	4221	13144	14658	SMARCA4
10255	8854	13292	6467	14247	4371	SMARCC1

IP: BICRA

Replicate #1		Replicate #2		Replicate #3		
BRD9 regular	BRD9 alternative	BRD9 regular	BRD9 alternative	BRD9 regular	BRD9 alternative	
10964	16670	17013	19486	16908	12342	BICRA
20355	5599	19057	6200	24807	6414	V5-tag
10987	10450	8183	6025	12291	7722	SMARCA4
8481	9902	3265	12345	10835	5391	SMARCC1

$$\text{Ratio} = \frac{\text{V5-tag (BRD9 regular)} \times \text{Protein (BRD9 alternative)}}{\text{V5-tag (BRD9 alternative)} \times \text{Protein (BRD9 regular)}}$$

E.g. BICRA $\text{Ratio} = \frac{31912 \times 25160}{5407 \times 36686} = 4.05$

$$\text{Ratio} = \frac{\text{BICRA (BRD9 regular)} \times \text{Protein (BRD9 alternative)}}{\text{BICRA (BRD9 alternative)} \times \text{Protein (BRD9 regular)}}$$

E.g. V5-tag BRD9 $\text{Ratio} = \frac{10964 \times 5599}{16670 \times 20355} = 0.18$

Supplementary Figure 13. *Agarose gel electrophoresis and Western blot images with quantification of protein expression levels and calculation of co-immunoprecipitation efficiency.*

A. Agarose gel electrophoresis image utilized in Figure 3E and G, alongside Western blot images of the BRD9 splice isoform analysis and co-immunoprecipitation experiments depicted in Figure 3I and Figure 4F, respectively. Blotting order is indicated by numbers. **B.** Quantification of protein expression levels for V5-tagged BRD9 splice isoforms, BICRA, SMARCA4, and SMARCC1 as shown in Figure 4F, and calculation of co-immunoprecipitation efficiency displayed in Figure 4G.

***Appendix 1**. DNA and protein sequence from two cloned BRD9 isoforms corresponding to BRD9 with the regular and the alternative C-terminus.*

The cloned regular *BRD9* splice variant shares 99.83% identity with NP_076413.3 (encoded by NM_023924.5), while the alternative *BRD9* splice variant shares 96.51% identity with XP_024301963.1 (encoded by XM_024446195.2). The DNA sequences are provided in FASTA format and named accordingly. The regular and alternative *BRD9* C-terminus sequences are highlighted in blue or red bold text, respectively. Corresponding protein sequences are shown below with the corresponding naming of the isoforms as for the transcripts.

```
>BRD9_REGULAR_C_TERMINUS_DNA
ATGGGCAAGAAGCACAAGAAGCACAAGGCCGAGTGGCGCTCGTCCTACGAGGATTATGCCGACAAGCCCCTGGAG
AAGCCTCTAAAGCTAGTCCTGAAGGTCGGAGGAAGTGAAGTGACTGAACTCTCAGGATCCGGCCACGACTCCAGT
TACTATGATGACAGGTCAGACCATGAGCGAGAGAGGCACAAAGAAAAGAAAAAGAAGAAGAAGAAGAAGTCCGAG
AAGGAGAAGCATCTGGACGATGAGGAAAGAAGGAAGCGAAAGGAAGAGAAGAAGCGGAAGCGAGAGAGGGAGCAC
TGTGACACGGAGGGAGAGGCTGACGACTTTGATCCTGGGAAGAAGGTGGAGGTGGAGCCGCCCCCAGATCGGCCA
GTCCGAGCGTGCCGGACACAGCCAGCCGAAAATGAGAGCACACCTATTCAGCAACTCCTGGAACACTTCCTCCGC
CAGCTTCAGAGAAAAGATCCCCATGGATTTTTTGCTTTTCCTGTCACGGATGCAATTGCTCCTGGATATTCAATG
ATAATAAAACATCCCATGGATTTTGGCACCATGAAAGACAAAATTGTAGCTAATGAATACAAGTCAGTTACGGAA
TTTAAGGCAGATTTCAAGCTGATGTGTGATAATGCAATGACATACAATAGGCCAGATACCGTGTACTACAAGTTG
GCGAAGAAGATCCTTCACGCAGGCTTTAAGATGATGAGCAAACAGGCAGCTCTTTTGGGCAATGAAGATACAGCT
GTTGAGGAACCTGTCCCTGAAGTTGTACCAGTACAAGTAGAAACTGCCAAGAAATCCAAAAAGCCGAGTAGAGAA
GTTATCAGCTGCATGTTTGAGCCTGAAGGGAATGCCTGCAGCTTGACGGACAGTACCGCAGAGGAGCACGTGCTG
GCGCTGGTGGAGCACGCAGCTGACGAAGCTCGGGACAGGATCAACCGGTTCCTCCCAGGCGGCAAGATGGGCTAT
CTGAAGAGGAACGGGGACGGGAGCCTGCTCTACAGCGTGGTCAACACGGCCGAGCCGGACGCTGATGAGGAGGAG
ACCCACCCGGTGGACTTGAGCTCGCTCTCCAGTAAGCTACTCCCAGGCTTCACCACGCTGGGCTTCAAAGACGAG
AGAAGAAACAAAGTCACCTTTCTCTCCAGTGCCACTACTGCGCTTTCGATGCAGAATAATTCAGTATTTGGCGAC
TTGAAGTCGGACGAGATGGAGCTGCTCTACTCAGCCTACGGAGATGAGACAGGCGTGCAGTGTGCGCTGAGCCTG
CAGGAGTTTGTGAAGGATGCTGGGAGCTACAGCAAGAAAGTGGTGGACGACCTCCTGGACCAGATCACAGGCGGA
GACCACTCTAGGACGCTCTTCCAGCTGAAGCAGAGAAGAAATGTTCCCATGAAGCCTCCAGATGAAGCCAAGGTT
GGGGACACCCTAGGAGACAGCAGCAGCTCTGTTCTGGAGTTCATGTCGATGAAGTCCTATCCCGACGTTTCTGTG
GATATCTCCATGCTCAGCTCTCTGGGGAAGGTGAAGAAGGAGCTGGACCCTGACGACAGCCATTTGAACTTGGAT
GAGACGACGAAGCTCCTGCAGGACCTGCACGAAGCACAGGCGGAGCGCGGCGGCTCTCGGCCGTCGCCCAACCTC
AGCTCCCTGTCCAACGCCTCCGAGAGGGACCAGCACCACCTGGGAAGCCCTTCTCGCCTGAGTGTCGGGGAGCAG
CCAGACGTCACCCATGACCCCTATGAGTTTCTTCAGTCTCCAGAGCCTGCGGCCTCTGCCAAGACCTAA

>BRD9_ALTERNATIVE_C_TERMINUS_DNA
ATGGGCAAGAAGCACAAGAAGCACAAGGCCGAGTGGCGCTCGTCCTACGAGGATTATGCCGACAAGCCCCTGGAG
AAGCCTCTAAAGCTAGTCCTGAAGGTCGGAGGAAGTGAAGTGACTGAACTCTCAGGATCCGGCCACGACTCCAGT
TACTATGATGACAGGTCAGACCATGAGCGAGAGAGGCACAAAGAAAAGAAAAAGAAGAAGAAGAAGAAGTCCGAG
AAGGAGAAGCATCTGGACGATGAGGAAAGAAGGAAGCGAAAGGAAGAGAAGAAGCGGAAGCGAGAGAGGGAGCAC
TGTGACACGGAGGGAGAGGCTGACGACTTTGATCCTGGGAAGAAGGTGGAGGTGGAGCCGCCCCCAGATCGGCCA
GTCCGAGCGTGCCGGACACAGCCAGCCGAAAATGAGAGCACACCTATTCAGCAACTCCTGGAACACTTCCTCCGC
CAGCTTCAGAGAAAAGATCCCCATGGATTTTTTGCTTTTCCTGTCACGGATGCAATTGCTCCTGGATATTCGATG
ATAATAAAACATCCCATGGATTTTGGCACCATGAAAGACAAAATTGTAGCTAATGAATACAAGTCAGTTACGGAA
TTTAAGGCAGATTTCAAGCTGATGTGTGATAATGCAATGACATACAATAGGCCAGATACCGTGTACTACAAGTTG
GCGAAGAAGATCCTTCACGCAGGCTTTAAGATGATGAGCAAACAGGCAGCTCTTTTGGGCAATGAAGATACAGCT
GTTGAGGAACCTGTCCCTGAAGTTGTACCAGTACAAGTAGAAACTGCCAAGAAATCCAAAAAGCCGAGTAGAGAA
GTTATCAGCTGCATGTTTGAGCCTGAAGGGAATGCCTGCAGCTTGACGGACAGTACCGCAGAGGAGCACGTGCTG
GCGCTGGTGGAGCACGCAGCTGACGAAGCTCGGGACAGGATCAACCGGTTCCTCCCAGGCGGCAAGATGGGCTAT
CTGAAGAGGAACGGGGACGGGAGCCTGCTCTACAGCGTGGTCAACACGGCCGAGCCGGACGCTGATGAGGAGGAG
```

ACCCACCCGGTGGACTTGAGCTCGCTCTCCAGTAAGCTACTCCCAGGCTTCACCACGCTGGGCTTCAAAGACGAG
AGAAGAAACAAAGTCACCTTTCTCTCCAGTGCCACTACTGCGCTTTCGATGCAGAATAATTCAGTATTTGGCGAC
TTGAAGTCGGACGAGATGGAGCTGCTCTACTCAGCCTACGGAGATGAGACAGGCGTGCAGTGTGCGCTGAGCCTG
CAGGAGTTTGTGAAGGATGCTGGGAGCTACAGCAAGAAAGTGGTGGACGACCTCCTGGACCAGATCACAGGCGGA
GACCACTCTAGGACGCTCTTCCAGCTGAAGCAGAGAAGAAATGTTCCCATGAAGCCTCCAGATGAAGCCAAGGTT
GGGGACACCCTAGGAGACAGCAGCAGCTCTGTTCTGGAGTTCATGTCGATGAAGTCCTATCCCGACGTTTCTGTG
GATATCTCCATGCTCAGCTCTCTGGTTTCTTTCAGGGCCTGCCATCTATCACGTGATGCTGTACTAAAGTCGGAT
CGGAATTTGGTGTCTTATTACCACGGAGTCTGCTTTGTCAACCTTCAGATTTCTGTTTTAAAGTTAATGCTGGTC
AGCTGTGCCTGA

>BRD9_REGULAR_C_TERMINUS_PROTEIN
MGKKHKKHKAEWRSSYEDYADKPLEKPLKLVLKVGGSEVTELSGSGHDSSYYDDRSDHERERHKEKKKKKKKSE
KEKHLDDEERRKRKEEKKRKREREHCDTEGEADDFDPGKKVEVEPPPDRPVRACRTQPAENESTPIQQLLEHFLR
QLQRKDPHGFFAFPVTDAIAPGYSMIIKHPMDFGTMKDKIVANEYKSVTEFKADFKLMCDNAMTYNRPDTVYYKL
AKKILHAGFKMMSKQAALLGNEDTAVEEPVPEVVPVQVETAKKSKKPSREVISCMFEPEGNACSLTDSTAEEHVL
ALVEHAADEARDRINRFLPGGKMGYLKRNGDGSLLYSVVNTAEPDADEEETHPVDLSSLSSKLLPGFTTLGFKDE
RRNKVTFLSSATTALSMQNNSVFGDLKSDEMELLYSAYGDETGVQCALSLQEFVKDAGSYSKKVVDDLLDQITGG
DHSRTLFQLKQRRNVPMKPPDEAKVGDTLGDSSSSVLEFMSMKSYPDVSVDISMLSSLGKVKKELDPDDSHLNLD
ETTKLLQDLHEAQAERGGSRPSPNLSSLSNASERDQHHLGSPSRLSVGEQPDVTHDPYEFLQSPEPAASAKT

>BRD9_ALTERNATIVE_C_TERMINUS_PROTEIN
MGKKHKKHKAEWRSSYEDYADKPLEKPLKLVLKVGGSEVTELSGSGHDSSYYDDRSDHERERHKEKKKKKKKSE
KEKHLDDEERRKRKEEKKRKREREHCDTEGEADDFDPGKKVEVEPPPDRPVRACRTQPAENESTPIQQLLEHFLR
QLQRKDPHGFFAFPVTDAIAPGYSMIIKHPMDFGTMKDKIVANEYKSVTEFKADFKLMCDNAMTYNRPDTVYYKL
AKKILHAGFKMMSKQAALLGNEDTAVEEPVPEVVPVQVETAKKSKKPSREVISCMFEPEGNACSLTDSTAEEHVL
ALVEHAADEARDRINRFLPGGKMGYLKRNGDGSLLYSVVNTAEPDADEEETHPVDLSSLSSKLLPGFTTLGFKDE
RRNKVTFLSSATTALSMQNNSVFGDLKSDEMELLYSAYGDETGVQCALSLQEFVKDAGSYSKKVVDDLLDQITGG
DHSRTLFQLKQRRNVPMKPPDEAKVGDTLGDSSSSVLEFMSMKSYPDVSVDISMLSSLVSFRACHLSRDAVLKSD
RNLVSYYHGVCFVNLQISVLKLMLVSCA
